# The spliceosome impacts morphogenesis in the human fungal pathogen *Candida albicans*

**DOI:** 10.1128/mbio.01535-24

**Published:** 2024-07-09

**Authors:** Emma Lash, Corinne Maufrais, Guilhem Janbon, Nicole Robbins, Lydia Herzel, Leah E. Cowen

**Affiliations:** 1 Department of Molecular Genetics, University of Toronto, Toronto, Ontario, Canada; 2 Unité Biologie des ARN des Pathogènes Fongiques, Institut Pasteur, Université Paris Cité, Paris, France; 3 HUB Bioinformatique et Biostatistique, Institut Pasteur, Université Paris Cité, Paris, France; 4 Institute of Chemistry and Biochemistry, Freie Universität Berlin, Berlin, Germany; Biomedicine Discovery Institute, Monash University, Clayton, Victoria, Australia

**Keywords:** *Candida albicans*, fungal pathogen, morphogenesis, temperature, spliceosome, intron, filamentation

## Abstract

At human body temperature, the fungal pathogen *Candida albicans* can transition from yeast to filamentous morphologies in response to host-relevant cues. Additionally, elevated temperatures encountered during febrile episodes can independently induce *C. albicans* filamentation. However, the underlying genetic pathways governing this developmental transition in response to elevated temperatures remain largely unexplored. Here, we conducted a functional genomic screen to unravel the genetic mechanisms orchestrating *C. albicans* filamentation specifically in response to elevated temperature, implicating 45% of genes associated with the spliceosome or pre-mRNA splicing in this process. Employing RNA-Seq to elucidate the relationship between mRNA splicing and filamentation, we identified greater levels of intron retention in filaments compared to yeast, which correlated with reduced expression of the affected genes. Intriguingly, homozygous deletion of a gene encoding a spliceosome component important for filamentation (*PRP19*) caused even greater levels of intron retention compared with wild type and displayed globally dysregulated gene expression. This suggests that intron retention is a mechanism for fine-tuning gene expression during filamentation, with perturbations of the spliceosome exacerbating this process and blocking filamentation. Overall, this study unveils a novel biological process governing *C. albicans* filamentation, providing new insights into the complex regulation of this key virulence trait.

**IMPORTANCE:**

Fungal pathogens such as *Candida albicans* can cause serious infections with high mortality rates in immunocompromised individuals. When *C. albicans* is grown at temperatures encountered during human febrile episodes, yeast cells undergo a transition to filamentous cells, and this process is key to its virulence. Here, we expanded our understanding of how *C. albicans* undergoes filamentation in response to elevated temperature and identified many genes involved in mRNA splicing that positively regulate filamentation. Through transcriptome analyses, we found that intron retention is a mechanism for fine-tuning gene expression in filaments, and perturbation of the spliceosome exacerbates intron retention and alters gene expression substantially, causing a block in filamentation. This work adds to the growing body of knowledge on the role of introns in fungi and provides new insights into the cellular processes that regulate a key virulence trait in *C. albicans*.

## INTRODUCTION

While the fungal kingdom boasts an incredible number of diverse species, only a select subset are capable of infecting humans and causing either superficial infections, such as athlete’s foot, or invasive disease that can spread to vital internal organs resulting in sepsis and death. Luckily for humans, most fungal species are thwarted by our mucosal barriers, immune system, and physiological body temperature, which prevent potential pathogens from causing systemic disease. However, the deployment of immunosuppressive regimens in modern medical practices and immune suppression due to infection with HIV-1 have resulted in a dramatic surge in vulnerable individuals over recent years, and consequently, fungal pathogens are now a leading cause of human mortality, collectively resulting in ~2.5 million deaths annually (1). A leading contributor to human mortality is *Candida albicans,* an opportunistic fungus that exists as a commensal member of the human microbiota until an event such as host immunosuppression provides the opportunity for escape and infection. Based on criteria such as rates of incidence, fatality, and antifungal drug resistance, the World Health Organization recently classified *C. albicans* as a critical threat pathogen (2).

The success of *C. albicans* as both a commensal and pathogen relies on its ability to sense and appropriately respond to changes in the environment. One of the most notable *C. albicans* virulence traits that requires acute environmental sensing is morphogenesis, which is the ability to switch between unicellular yeast and multicellular filamentous growth states. Most mutants that are locked in either state are avirulent in mouse models of systemic candidiasis (3, 4). Many *in vitro* studies have been devoted to understanding the signaling pathways that allow *C. albicans* to sense and respond to different filament-inducing stimuli, which include blood serum (5), elevated carbon dioxide (6), nutrient starvation (7), and macrophage internalization (5, 8). Most inducing cues signal through the cyclic AMP (cAMP) protein kinase A (PKA) pathway, in which a rise in intracellular cAMP triggers the activation of the PKA complex, which phosphorylates transcription factors such as Efg1 to upregulate the expression of hyphal-specific genes (9). However, in order for these signaling cascades to induce robust filamentous growth, a concurrent temperature increase to at least 37°C is required (10). Robust filamentation is also induced when *C. albicans* is grown at or above 39°C, and as such, high temperature represents its own independent inducing cue (10). Given that patient body temperature can surpass these thresholds during febrile episodes, it is critical that we understand how temperature impacts this key virulence trait.

Thermotolerance and temperature-dependent filamentation are inextricably linked through the molecular chaperone Hsp90 (11, 12). Previous work established that Hsp90 is a repressor of morphogenesis such that at high temperatures, when the functional capacity of the chaperone is overwhelmed by the accumulation of misfolded proteins, its repression on PKA signaling is relieved, thus enabling filamentous growth (13). This is also shown by the fact that pharmacological inhibition of Hsp90 or genetic depletion of *HSP90* results in the induction of filamentation independent of elevated temperature (13). Furthermore, Hsp90’s function in protein homeostasis is essential for thermotolerance (11, 12). Thus, Hsp90 links morphogenesis and thermotolerance, but other genetic pathways remain to be explored.

Functional and chemical genomic studies have been instrumental in discovering cellular processes that allow *C. albicans* to switch between distinct morphological states. For example, endocytosis is critical for filamentation as it is needed to recycle membranes and membrane proteins at the growing hyphal tip (14). As such, compounds that impede endocytic trafficking or genetic deletion of endocytic components, such as *RVS161*, *RVS167*, and *ENT2*, result in a block in filamentation (15–17). Additionally, a functional genomic screen of a repressible *C. albicans* mutant library found a key role for ergosterol biosynthesis in regulating filamentation, which was further supported by the fact that sublethal concentrations of the antifungal drug fluconazole, which targets ergosterol biosynthesis, also blocks filamentation (5). Finally, genetic analyses have revealed that proper actin organization is critical for filamentation, both for the delivery of secretory vesicles along actin cables and for the formation of actin patches required for endocytosis (10). Specifically, treatment with the actin-depolymerizing drugs cytochalasin A or latrunculin A inhibits morphogenesis and suppresses the expression of the hyphal-specific gene *HWP1* (18). Overall, these approaches have uncovered diverse cellular processes that govern morphogenesis, but our understanding of the underlying biological complexity is still incomplete.

The spliceosome is a large, dynamic RNA-protein complex that coordinates the removal of introns to produce spliced mRNA through a two-step transesterification reaction. In *Saccharomyces cerevisiae,* over 100 proteins are involved in mRNA splicing, including those that form complexes with the five small nuclear RNAs (snRNAs) to generate small nuclear ribonucleoproteins (snRNPs) and non-snRNP auxiliary proteins, which help catalyze the process (19). The highly dynamic conformation of the spliceosome not only allows for precise splicing of diverse substrates but also offers opportunities for regulation in response to environmental changes (20). In fungi, the most common form of alternative splicing is intron retention (IR), whereby an intron is selectively retained in the final mRNA product (21). Though only ~5% of *S. cerevisiae* genes possess an intron, regulation of their splicing can impact the organisms’ ability to adapt to environmental changes (22–24). For example, in the model yeast, different sets of transcripts are subject to reduced splicing when exposed to two unrelated environmental stresses, amino acid starvation and ethanol toxicity (20). Furthermore, intron-mediated regulation of the ribosomal protein gene, *RPS22B*, induces phenotypic heterogeneity in *S. cerevisiae* in response to nutrient starvation, which allows cells to adapt to a changing environment (25). Overall, this suggests that introns have been selectively retained in the *S. cerevisiae* genome as a mechanism to promote cell survival in variable environments. The spliceosome is well conserved among eukaryotes, especially between *S. cerevisiae* and *C. albicans* (26). Also, the gene architecture of intron-containing genes, with mainly single introns that are within the 5′ portion of the transcript, is conserved between *S. cerevisiae* and *C. albicans* (27). Despite only ~6% of *C. albicans* genes possessing an intron, examples of alternative splicing have been explored in this organism (28–31). Furthermore, the enrichment of specific biological processes, such as ribosome assembly, translation, proton motive force-driven ATP synthesis, and RNA binding among intron-containing genes suggests that introns play an important role in *C. albicans,* which begs for further exploration (Table S1) (28, 31).

In this work, we performed a functional genomic screen to identify positive regulators of *C. albicans* filamentation induced by growth at high temperatures. Through this analysis, we identified 38 genes for which genetic depletion resulted in a filamentation defect without substantially affecting growth, many of which are important for mRNA splicing. From here, we built a mutant collection to cover every *C. albicans* homolog of an *S. cerevisiae* spliceosome component and provided an in-depth characterization of their role in filamentation. Furthermore, we performed RNA-Seq to map splicing and expression changes in wild-type yeast and filaments, as well as in a spliceosome mutant that is unable to filament, and detect IR as a complex fine-tuning modulator for gene expression that regulates morphogenesis. Overall, this work links a core cellular process, mRNA splicing, to a key virulence trait in the human fungal pathogen *C. albicans*.

## RESULTS

### Functional genomic screen identifies genes required for filamentation at elevated temperature

**Fig 1:**
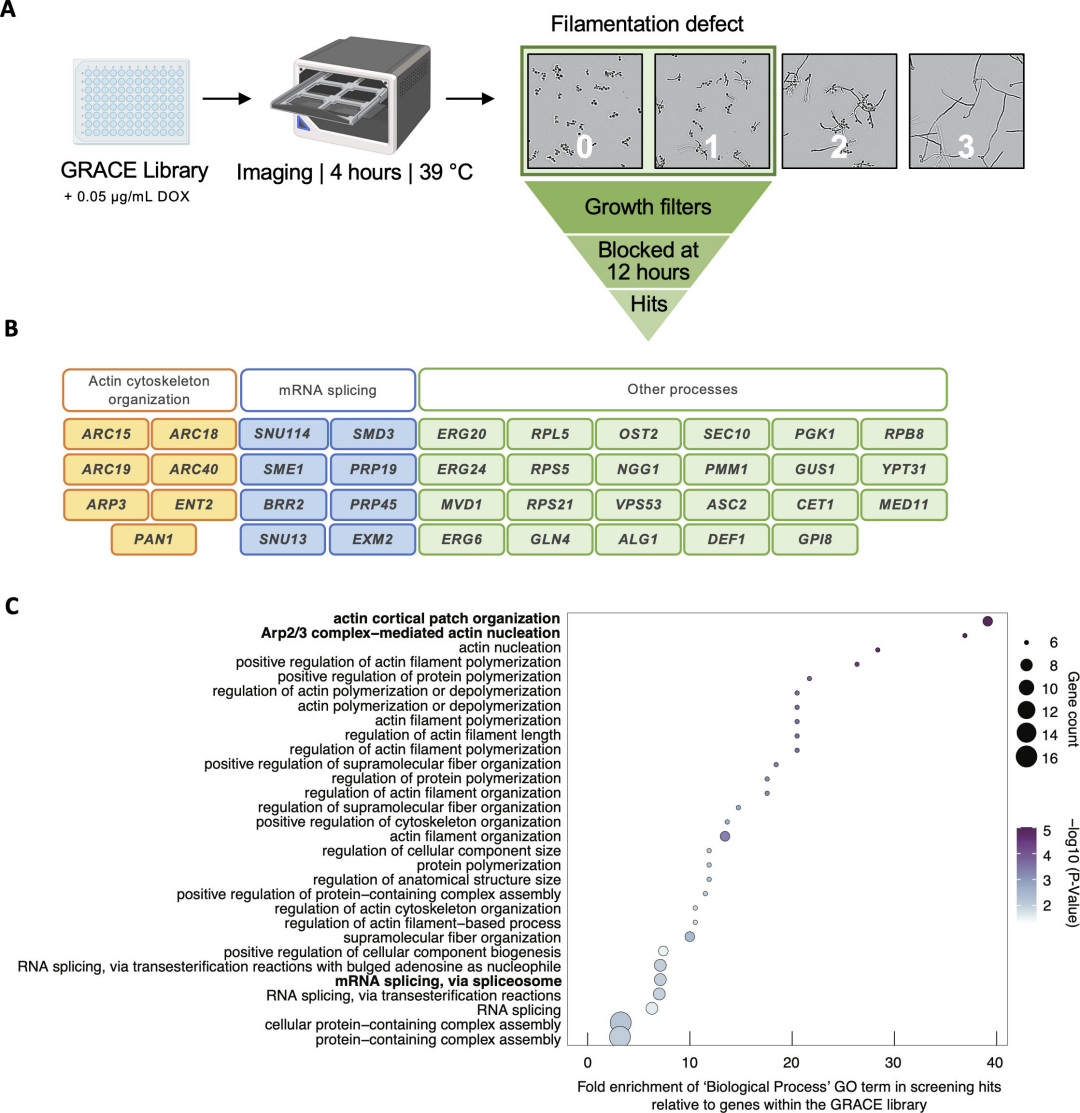
Functional genomic screen of the GRACE library identifies 38 genes important for filamentation at 39°C. (**A**) Schematic of the functional genomic screen of the GRACE library to identify mutants blocked in filamentation at 39°C. Each strain was treated with 0.05 µg/mL DOX and grown overnight and then diluted into the same conditions the following day to repress target gene expression. Filamentation was scored after 4 hours of static growth at 39°C on a scale of 0–3, where 3 indicates strains that filament to the same extent as the wild-type strain and 0 indicates a complete block in filamentation. Growth was assessed after 24 hours at 30°C (OD_600_) and 39°C (visual inspection of images), and strains with a severe growth defect were filtered out. Candidate hits were also examined for their ability to filament at 12 hours, with those that maintained a block in filamentation being prioritized for further analysis. Images were taken using the IncuCyte S3 Live-Cell Analysis at 10× magnification. (**B**) Thirty-eight genes identified as being important for high temperature-induced filamentation. Genes involved in actin cytoskeleton organization are colored in yellow, genes involved in mRNA splicing are colored in blue, and genes involved in other processes are colored in green. (**C**) Dot plot of significantly enriched GO biological process terms among screening hits. The most specific significantly enriched GO terms are bolded.

Functional genomic analyses have provided valuable insights into the genetic mechanisms underlying filamentation in response to various inducing cues such as serum, Hsp90 inhibition, and phagocytosis by macrophages (5, 8, 32). However, a systematic genetic analysis of the genes important for filamentation in response to elevated temperatures has yet to be performed. To address this gap, we utilized the original gene replacement and conditional expression (GRACE) collection, which consists of 2,356 mutants in which one allele of a gene of interest is deleted, and the remaining allele is controlled by a tetracycline-repressible promoter (33). Thus, the addition of tetracycline or its analog doxycycline (DOX) enables transcriptional repression of the remaining allele to interrogate loss-of-function phenotypes. To perform the screen, each GRACE strain was grown overnight in rich medium (YPD) supplemented with 0.05 µg/mL DOX at 30°C. The following day, strains were sub-cultured into YPD containing 0.05 µg/mL DOX and imaged after 4 hours of growth at 39°C (Fig. 1A). Each image was manually scored on a scale of 0–3, where 0 indicated a strain with a complete block in filamentation and 3 indicated a strain exhibiting wild-type levels of filamentation (Fig. 1A; Table S2). Strains that were completely blocked (score: 0) or showed substantial defects in filamentation (score: 1) were considered hits, identifying 116 genes important for filamentation in response to high temperature. To prioritize the most robust hits for mechanistic studies, strains with a severe growth defect, characterized by a reduction in growth of more than 75% relative to wild type at 24 hours at either 30°C (measured by OD_600_) or 39°C (assessed by manually inspecting images), were removed to ensure defects in morphogenesis were not confounded by impaired growth (Table S2). In addition, strains for which filamentation was restored after growth at elevated temperature for 12 hours were removed from our prioritized hit list (Fig. S1A). This comprehensive analysis revealed 38 genes that are required for filamentation at 39°C (Fig. 1B). Notably, several of these genes have previously been implicated in filamentation, such as those involved in ergosterol biosynthesis (*ERG6, ERG20,* and *ERG24*) (5), the Golgi-associated retrograde protein complex (*VPS53*) (34)*,* and the Arp2/3 complex (*ARC15, ARC19, ARC18, ARC40,* and *ARP3*) (35). Moreover, a significant enrichment of biological processes related to actin organization was observed among the hits (Fig. 1C). Interestingly, biological processes involved in mRNA splicing also showed significant enrichment (Fig. 1C). To the best of our knowledge, no connection between mRNA splicing and filamentation in *C. albicans* has been established, thus prompting further investigation.

### Genes involved in mRNA splicing via the spliceosome are important for filamentation

Given the intriguing observation from our primary screen, we sought to attain a more comprehensive assessment as to whether genes involved in this process are important for filamentation, as only 57 genes out of 97 predicted to have roles in mRNA splicing are present in the original GRACE library. To accomplish this, we generated GRACE strains for all genes annotated with the Gene Ontology (GO) term “mRNA splicing via the spliceosome” in the *Candida* Genome Database (CGD) (26). Additionally, to ensure comprehensive coverage, we generated GRACE mutants for *C. albicans* homologs of *S. cerevisiae* genes annotated with this GO term as identified from the *Saccharomyces* Genome Database (SGD) (36). This list was subsequently manually curated to ensure every gene had a true “mRNA splicing” GO term annotation in either CGD or SGD. This guided the generation of an additional 38 GRACE strains (37) (see Materials and Methods), enabling an in-depth analysis of a set of 95 mutants.

**Fig 2:**
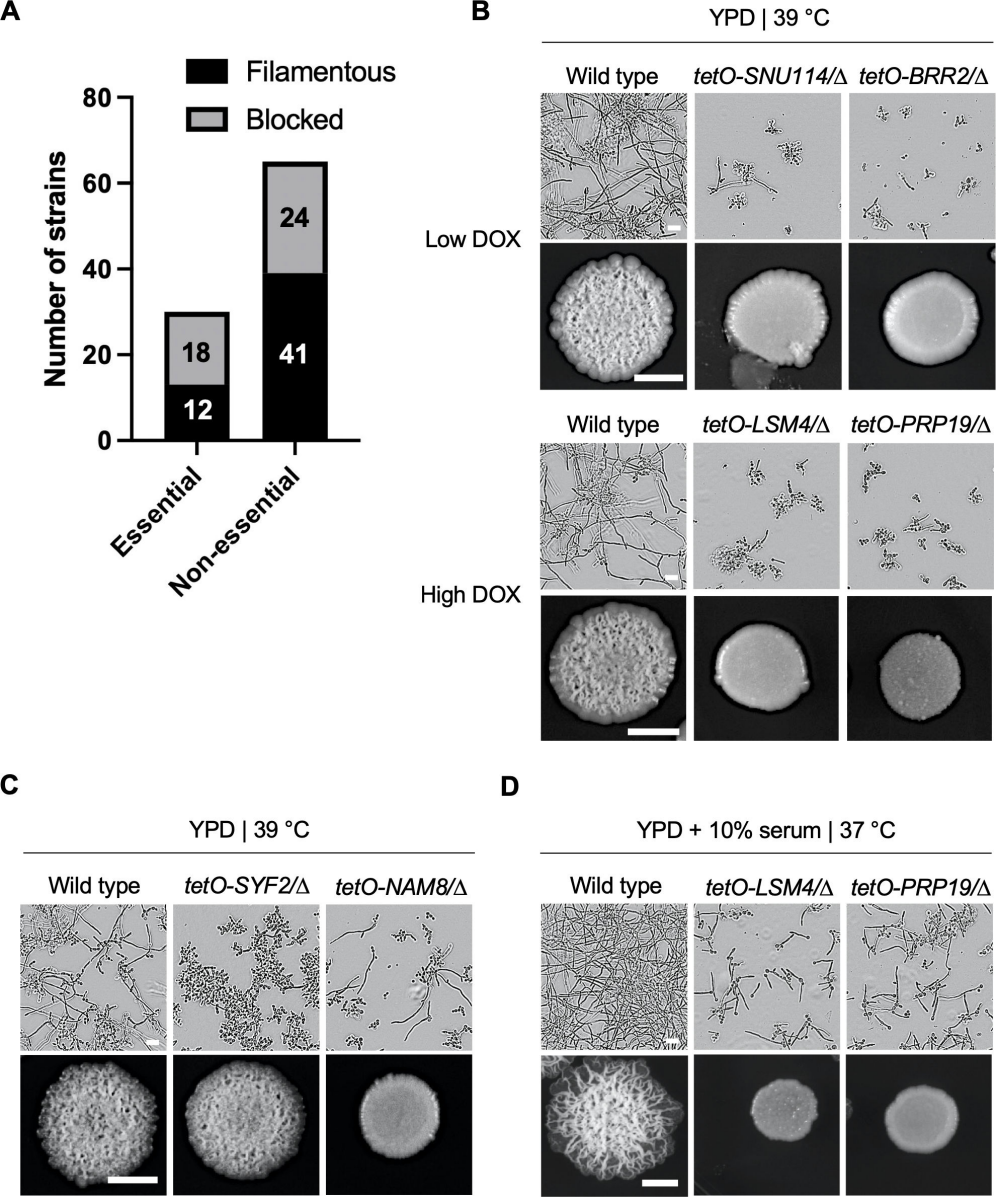
The spliceosome is important for filamentation at 39°C in liquid and solid media. (**A**) The number of essential and non-essential GRACE spliceosome mutants is shown. If depletion of the target gene results in a filamentation defect (score of 0 or 1) in at least one condition (Table 1), it is indicated as blocked. If no defect is observed in any condition tested, it is indicated as filamentous. (**B**) Representative GRACE spliceosome mutants that are blocked in filamentation in both liquid and solid. For liquid YPD (top), strains were grown for 6 hours in static conditions and then imaged. For solid YPD (bottom), strains were grown for 72 hours and then imaged. Low DOX = 0.1 µg/mL. High DOX = 20 µg/mL. All phenotypes were DOX dependent. (**C**) Representative GRACE spliceosome mutants that are blocked in filamentation in liquid or on solid, but not both. Phenotypes were assessed as in panel B. (**D**) Representative GRACE spliceosome mutants that are capable of filamenting in YPD supplemented with 10% serum at 37°C in liquid but not on solid. Phenotypes were assessed as in panel B. Scale bar for liquid images is 20 µm. Scale bar for solid images is 5 mm.

First, we examined the importance of all 95 genes for growth by assessing colony formation upon transcriptional repression using a very high concentration of DOX (100 µg/mL). This revealed 30 essential and 65 non-essential genes among this set (Fig. 2A; Table S2). Next, we investigated all strains under low DOX (0.05–0.1 µg/mL) and high DOX (20 µg/mL) conditions to observe morphogenesis phenotypes associated with partial repression of essential genes and complete repression of non-essential genes, respectively (Fig. S1B). This revealed 30 genes involved in “mRNA splicing via the spliceosome” that are important for filamentation in liquid YPD at 39°C (Table 1; Fig. 2B). Interestingly, most mutants that were blocked in filamentation in liquid YPD at 39°C were also unable to form wrinkly colonies after 72 hours on solid YPD agar at 39°C (Fig. 2B; Table 1). Only five strains that were blocked in filamentation in liquid were capable of wrinkly colony formation (Table 1). Furthermore, an additional 12 mutants were blocked in wrinkly colony formation but capable of forming filaments in liquid (Table 1). As an example, the *tetO-SYF2/syf2∆* strain was capable of forming wrinkly colonies on solid but did not undergo filamentous growth in liquid in the presence of DOX (Fig. 2C). In contrast, the *tetO-NAM8/nam8∆* strain displayed the opposite phenotype such that filamentation was observed in liquid medium, but smooth colonies formed on solid agar in the presence of DOX (Fig. 2C). These findings add to the growing body of evidence that both distinct and overlapping genetic components govern morphogenesis in liquid and on solid, even in response to the same inducing stimulus (38, 39). Among the genes with a role in filamentation, 18 were essential and 24 were non-essential (Fig. 2A; Table 1).

**TABLE 1:** Spliceosome genes required for filamentation^*a*^

Common name/ homolog	Feature name	Description	Liquid 39°C	Solid 39°C	Liquid serum	Solid serum	Essentiality
*BRR2*	*C4_07100C_A*	Predicted RNA-dependent ATPase RNA helicase	Yeast	Yeast	Filament	Yeast	E
*SNU114*	*C2_04640C_A*	Protein similar to *S. cerevisiae* Snu114p, which is an RNA helicase involved in pre-mRNA splicing	Yeast	Yeast	Filament	Yeast	E
*PRP19*	*C3_07480W_A*	Ortholog(s) have ubiquitin-protein transferase activity and role in the generation of catalytic spliceosome for first transesterification step	Yeast	Yeast	Filament	Yeast	NE
*PRP45*	*C6_02440C_A*	Protein required for pre-mRNA splicing	Yeast	Yeast	Yeast	Yeast	E
*SNU13*	*C3_04380C_A*	Putative U3 snoRNP protein	Yeast	Yeast	Filament	Yeast	E
*PRP42*	*CR_03750C_A*	Putative component of the U1 snRNP, involved in splicing	Yeast	Yeast	Filament	Yeast	E
*LSM3*	*CR_07330W_A*	Component of heteroheptameric complexes (Lsm1p and Lsm8p) involved in RNA processing and decay	Yeast	Yeast	Filament	Yeast	NE
*LSM5*	*C1_14410W_A*	Ortholog of *S. cerevisiae/Schizosaccharomyces pombe* Lsm5; Lsm (like Sm) protein involved in mRNA decay	Yeast	Filament	Filament	Filament	NE
*SMD1*	*CR_10820W_A*	Ortholog(s) have mRNA binding activity, role in mRNA splicing via spliceosome and U1 snRNP, U2-type prespliceosome, U4/U6 x U5 tri-snRNP complex, U5 snRNP, commitment complex, cytosol localization	Yeast	Yeast	Filament	Yeast	E
*PRP40*	*CR_01050C_A*	Ortholog(s) have RNA binding activity, role in mRNA splicing via spliceosome and U1 snRNP, U2-type prespliceosome localization	Yeast	Yeast	Filament	Yeast	NE
*SME1*	*C6_00530C_A*	Ortholog(s) have role in mRNA splicing via spliceosome and U1 snRNP, U2 snRNP, U2-type prespliceosome, U4/U6 x U5 tri-snRNP complex, U5 snRNP, post-mRNA release spliceosomal complex localization	Yeast	Yeast	Filament	Yeast	E
*MSL5*	*C3_03300C_A*	Putative pre-mRNA branch point binding protein; role in mRNA splicing via spliceosome	Yeast	Yeast	Filament	Yeast	E
*SMD3*	*C5_01510W_A*	Putative core snRNP protein	Yeast	Yeast	Filament	Yeast	E
*LSM2*	*CR_04260W_A*	Putative U6 snRNA-associated protein	Yeast	Yeast	Filament	Yeast	NE
*SYF1*	*C4_06410W_A*	Ortholog is member of the NineTeen Complex that contains Prp19p and stabilizes U6 snRNA in catalytic forms of the spliceosome containing U2, U5, and U6 snRNAs	Filament	Yeast	Filament	Filament	NE
*EXM2*	*C4_06930C_A*	Putative U1 snRNP complex component	Yeast	Yeast	Filament	Filament	E
*YHC1*	*C2_06300W_A*	Ortholog(s) have mRNA binding, pre-mRNA 5′-splice site binding activity, role in mRNA 5′-splice site recognition and U1 snRNP, U2-type prespliceosome, commitment complex localization	Filament	Yeast	Filament	Yeast	NE
*PRP24*	*CR_01700C_A*	Ortholog(s) have U6 snRNA binding activity, role in spliceosomal complex assembly, spliceosomal tri-snRNP complex assembly, and U6 snRNP localization	Filament	Yeast	Filament	Filament	E
*SNU66*	*C5_03010W_A*	Ortholog(s) have role in mRNA splicing via spliceosome, maturation of 5S rRNA, and U4/U6 x U5 tri-snRNP complex localization	Yeast	Yeast	Filament	Filament	E
*BUD31*	*C1_09880C_A*	Ortholog is component of the SF3b subcomplex of the U2 snRNP; increases efficiency of first and second step pre-mRNA splicing	Filament	Yeast	Filament	Filament	NE
*PRP18*	*C2_00200W_A*	snRNP U5 splicing factor component; involved in positioning the 3′ splice site during the second catalytic step of splicing	Filament	Yeast	Filament	Yeast	E
*NPL3*	*C1_14280C_A*	Putative RNA-binding protein	Filament	Yeast	Filament	Filament	NE
*SNP1*	*C2_05540C_A*	Putative U1-70K component of the U1 snRNP, involved in splicing	Yeast	Yeast	Filament	Yeast	NE
*HSH49*	*C2_07060W_A*	Ortholog(s) have RNA binding activity, role in mRNA splicing via spliceosome and U2 snRNP, U2-type prespliceosome localization	Yeast	Yeast	Filament	Yeast	NE
*SMB1*	*CR_07510W_A*	Ortholog(s) have role in mRNA splicing, via spliceosome and U1 snRNP, U2 snRNP, U2-type prespliceosome, U4/U6 x U5 tri-snRNP complex, U5 snRNP, post-mRNA release spliceosomal complex localization	Yeast	Yeast	Filament	Yeast	E
*SMD2*	*C2_06240W_A*	Putative core Sm protein	Yeast	Yeast	Filament	Yeast	NE
*CWC25*	*C5_04080C_A*	Putative splicing factor required for the first step of pre-mRNA splicing	Filament	Yeast	Filament	Yeast	NE
*SYF2*	*C7_04290W_A*	Ortholog(s) have role in mRNA splicing via spliceosome	Yeast	Filament	Filament	Filament	NE
*MUD2*	*C2_02770W_A*	Ortholog(s) have mRNA binding, pre-mRNA branch point binding activity, role in mRNA branch site recognition, mRNA *cis* splicing via spliceosome and U2-type prespliceosome, U2AF, commitment complex localization	Yeast	Yeast	Filament	Yeast	NE
*GCR3*	*C1_08420W*	Ortholog is mRNA-binding protein involved in splicing, nonsense-mediated decay, and response to osmotic stress; subunit of nuclear cap binding complex and also of spliceosomal commitment complex	Filament	Yeast	Filament	Yeast	NE
*PRP11*	*C1_08660C*	Ortholog(s) have RNA binding activity, role in spliceosomal complex assembly, and U2-type prespliceosome localization	Yeast	Filament	Filament	Filament	E
*SPT5*	*C2_01480W_A*	Protein similar to *S. cerevisiae* Spt5p transcription elongation factor	Yeast	Filament	Filament	Filament	E
*SPT4*	*C5_04630W_A*	Ortholog(s) have RNA polymerase II core binding, rDNA binding, single-stranded RNA binding activity	Yeast	Filament	Yeast	Filament	NE
*SMX2*	*C2_03880C_A*	Ortholog(s) have U1 snRNP, U2 snRNP, U2-type prespliceosome, U4/U6 x U5 tri-snRNP complex, U5 snRNP, post-mRNA release spliceosomal complex localization	Yeast	Yeast	Filament	Yeast	NE
*NAM8*	*C2_07520C_A*	Ortholog(s) have mRNA binding activity and role in mRNA splice site selection, mRNA splicing, via spliceosome, positive regulation of mRNA splicing, via spliceosome	Filament	Yeast	Filament	Yeast	NE
*PRP8*	*CR_08660W_A*	Protein similar to *S. cerevisiae* Prp8, a component of the U4/U6-U5 snRNP complex,	Yeast	Yeast	Yeast	Yeast	E
*HUB1*	*C4_05040W_A*	Ortholog is protein tag that contributes to messenger RNA (mRNA) splicing, cell morphogenesis involved in conjugation with cellular fusion, and protein modification	Filament	Yeast	Filament	Filament	NE
*LSM7*	*C5_03400C_A*	Lsm protein, predicted role in mRNA decay	Yeast	Yeast	Filament	Yeast	NE
*LSM4*	*C7_02510W_A*	Ortholog(s) have U6 snRNA binding activity and role in P-body assembly, mRNA splicing via spliceosome	Yeast	Yeast	Filament	Yeast	NE
*LSM6*	*CR_00240W_A*	Putative Lsm protein	Yeast	Yeast	Filament	Yeast	NE
*CEF1*	*C1_09370W_A*	Ortholog is essential splicing factor, associated with Prp19p and the spliceosome	Filament	Yeast	Filament	Filament	E
*PRP4*	*CR_09480W_A*	Ortholog(s) have role in snoRNA splicing, spliceosomal conformational changes to generate catalytic conformation, and U4/U6 x U5 tri-snRNP complex localization	Filament	Yeast	Filament	Yeast	NE

^*a*^ Genes required for filamentation at 39°C are shown. For filamentation, GRACE strains for essential genes were treated with 0.1 µg/mL DOX and GRACE strains for non-essential genes were treated with 20 µg/mL DOX. Liquid phenotypes were assessed after 6 hours of growth, and solid phenotypes were assessed after 72 hours of growth. 39°C = YPD. Serum = YPD + 10% newborn calf serum at 37°C. All assays were performed under static conditions. GRACE strains were treated with 100 µg/mL DOX for essentiality verdict, where E is essential and NE is non-essential.

To further investigate whether the spliceosome is specifically required for filamentation in response to elevated temperature, or has a more general role across various cues, we assessed the capacity of this set of mutants to filament in YPD supplemented with 10% serum at 37°C. Interestingly, most mutants were able to filament to some extent when cultured in liquid YPD with serum and DOX at 37°C but formed completely smooth colonies when grown on agar under the same conditions (Table 1; Fig. 2D). These more subtle defects observed in response to liquid YPD supplemented with serum could explain why this genetic signature had not been identified in previous GRACE screens (5).

### Actin is sufficiently spliced in the filament-defective spliceosome mutant, *prp19∆/∆*

Considering our finding that many spliceosome components are required for filamentation, the most plausible hypothesis is that transcripts encoding key proteins needed for filamentation require proper splicing and are inadequately spliced in these mutant backgrounds. Actin organization plays a critical role in filamentation, and genetic or pharmacological perturbations of this process can block filamentation (10). Interestingly, the gene encoding actin, *ACT1*, contains a 658 base-pair intron. Thus, we aimed to investigate whether a reduction in *ACT1* splicing could be responsible for the block in filamentation observed in these mutants.

**Fig 3:**
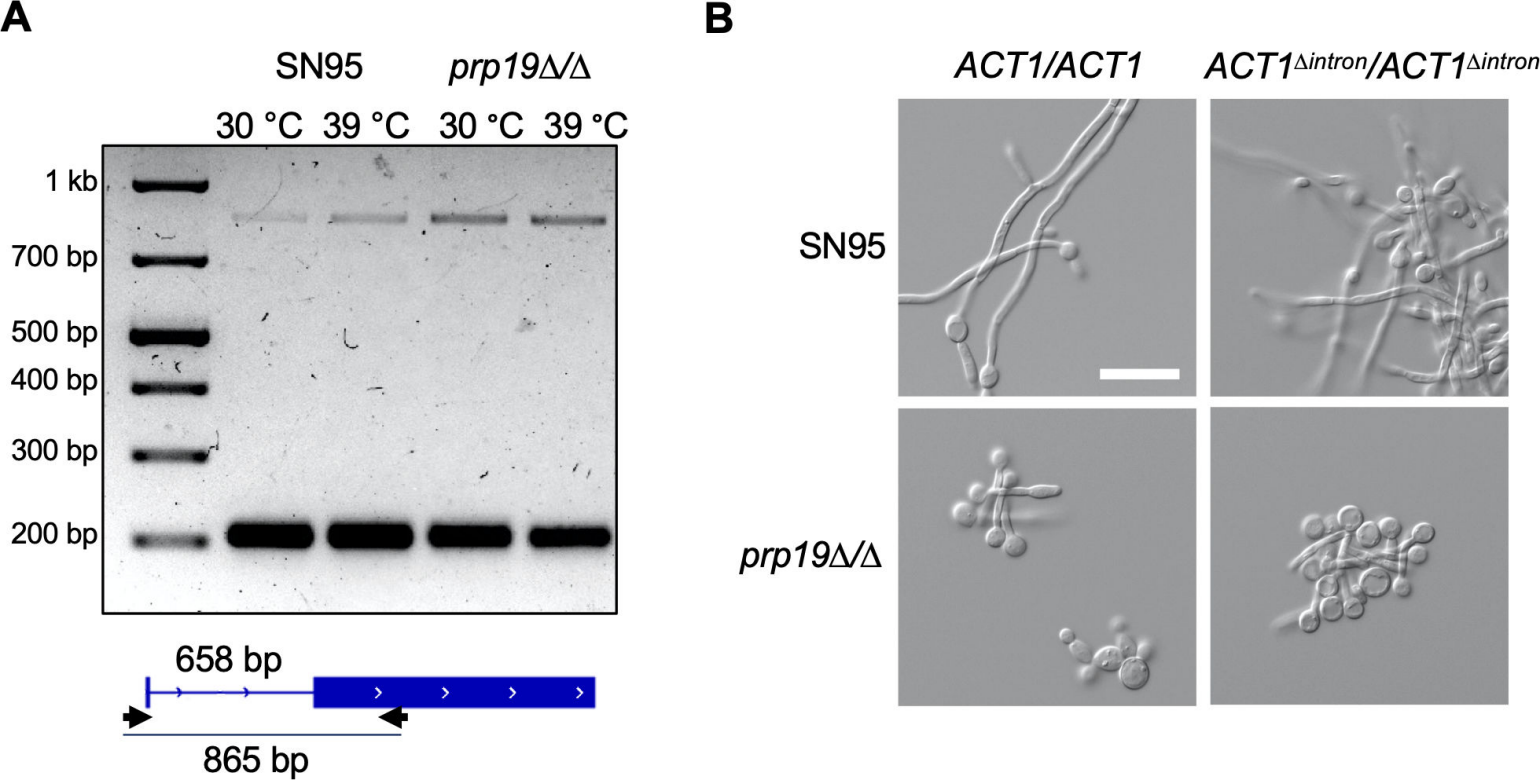
Failure to splice *ACT1* is not solely responsible for the block in filamentation of the *prp19∆/∆* mutant. (**A**) RT-PCR splicing assay for *ACT1*. Wild-type and *prp19∆/∆* cells were grown for 4 hours in YPD at 30°C or 39°C, RNA was extracted, cDNA was synthesized, and primers flanking the intron as shown (lower panel) were used to amplify the spliced and un-spliced products, as indicated by the marked DNA ladder. (**B**) Deletion of the *ACT1* intron does not impact filamentation. Strains were imaged after growth for 4 hours in YPD at 39°C, shaking. Scale bar is 20 µm.

To test this hypothesis, we deleted the gene encoding the spliceosome component Prp19. This mutant was selected as its *S. cerevisiae* homolog is a core component of the NineTeen complex, which is important for mRNA splicing; however, *PRP19* is non-essential, enabling the generation of a homozygous deletion mutant. We found that deletion of *PRP19* results in a strong defect in filamentous growth at 39°C (Fig. 3B). We performed a gel-based RT-PCR splicing assay on both wild-type and *prp19∆/∆* cells grown at 30°C and 39°C using *ACT1* primers spanning the intron to amplify both spliced and unspliced forms. Interestingly, while we observed a slight increase in band intensity corresponding to the size of the unspliced *ACT1* product in the *prp19∆/∆* mutant compared to wild type, we still detected a strong band corresponding to spliced *ACT1* similar to wild-type levels (Fig. 3A). To verify that the modest increase in unspliced *ACT1* and modest decrease in spliced *ACT1* did not exert a dominant negative effect on filamentation, we deleted the *ACT1* intron in both a wild-type strain and the *prp19∆/∆* mutant to see if this would rescue filamentous growth in the mutant background. However, removal of the *ACT1* intron failed to restore filamentation in the *prp19∆/∆* mutant (Fig. 3B), demonstrating that inefficient *ACT1* splicing is not solely responsible for the block in filamentation in this spliceosome mutant.

### Similar transcriptome profiles observed during *C. albicans* filamentation induced by different cues

As an alternative approach to providing mechanistic insights into how splicing governs filamentous growth, we decided to obtain a comprehensive global overview of splicing during filamentation in distinct inducing cues, including elevated temperature. To do this, we employed RNA-Seq to map splicing of all genes with introns in the *C. albicans* genome during filamentation induced by high temperature (39°C) or in response to serum at 37°C. We selected these two cues based on our observation that the filamentation phenotype of the GRACE spliceosome mutants was slightly different under these conditions (Table 1), and we were interested in whether these differences would be reflected in splicing patterns. For these experiments, wild-type (SN95) cells were grown in biological triplicate for 4 hours in liquid YPD at 30°C, 39°C, or YPD supplemented with 10% serum at 37°C before harvesting RNA for Illumina sequencing.

**Fig 4:**
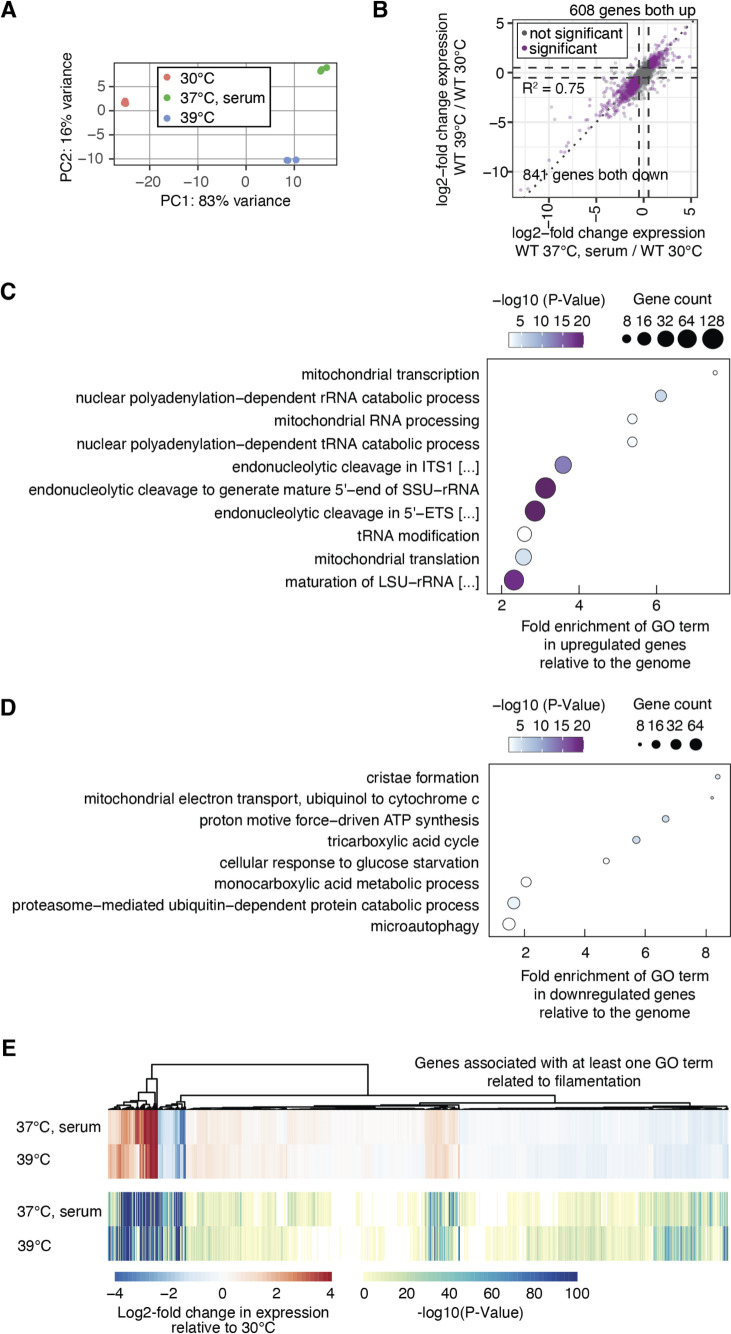
High temperature and serum-induced filaments have similar gene expression profiles. (**A**) Principal component analysis for three RNA-seq replicates per condition. (**B**) Scatter plot of log2-fold expression changes between the filament-inducing condition and growth at 30°C. Significant genes (shown in purple) have an absolute log2-fold change greater than 0.5 (dashed line) and adjusted *P*-value smaller than 0.00001. The dotted line reflects the diagonal. Gene expression analysis was done with DEseq2. (**C**) Significant GO terms (Process, Bonferroni-corrected *P*-value < 0.01) associated with upregulated genes in both filament-inducing conditions. (**D**) Significant GO terms (Process, Bonferroni-corrected *P*-value < 0.01) associated with downregulated genes in both filament-inducing conditions. (**E**) Heatmap of log2-fold changes in gene expression relative to growth at 30°C (upper panel) and heatmap of associated *P*-values for genes that are described by at least one GO term associated with filamentation (lower panel).

We employed DESeq2 to analyze the gene expression profiles of *C. albicans* filaments induced by growth at 39°C or in media supplemented with serum at 37°C (Table S3). Overall, there was great similarity among replicates, and significant differences were observed between yeast and filament conditions (Fig. 4A). Interestingly, filaments induced by the two different cues formed distinct clusters, indicating growth condition as a major source of variation in expression profile in addition to morphological state (Fig. 4A). Using an adjusted *P*-value cutoff of *P* < 0.00001 and requiring an absolute log2-fold change of 0.5, we found that under both filament-inducing conditions, 608 genes were upregulated, including *HWP1* and *ECE1* as the two most upregulated genes in both conditions, and 841 genes were downregulated compared to growth at 30°C out of 5,838 genes included in the analysis, which suggests that the transcriptional program is similar between filaments induced by these two conditions (Fig. 4B). Among the shared set of genes upregulated in filaments, there was a significant enrichment of biological processes relating to rRNA metabolism, ribosomal small subunit biogenesis, tRNA processing, and mitochondrial transcription/translation (Fig. 4C; Table S1). In contrast, genes that were downregulated in filaments were significantly enriched for terms related to aerobic respiration (Fig. 4D; Table S1). A subset of 10% of genes were differentially expressed between the two filament-inducing conditions and, interestingly, associated with distinct biological processes. Genes involved in ribosome biogenesis were more highly expressed in filaments induced by growth at 39°C relative to growth in serum at 37°C, whereas genes involved in copper and iron homeostasis had reduced expression at 39°C relative to serum at 37°C (Table S1). To assess how similar the filamentation program was between 39°C and serum, we investigated the differential expression of genes annotated to at least one GO term related to filamentation. Overall, genes in this category showed similar expression between the two conditions, indicating the gene regulatory program governing filamentation is similar between cells grown at 39°C and in media supplemented with serum at 37°C (Fig. 4E).

### Pre-mRNA splicing contributes to fine-tuning mRNA levels during filamentation

**Fig 5:**
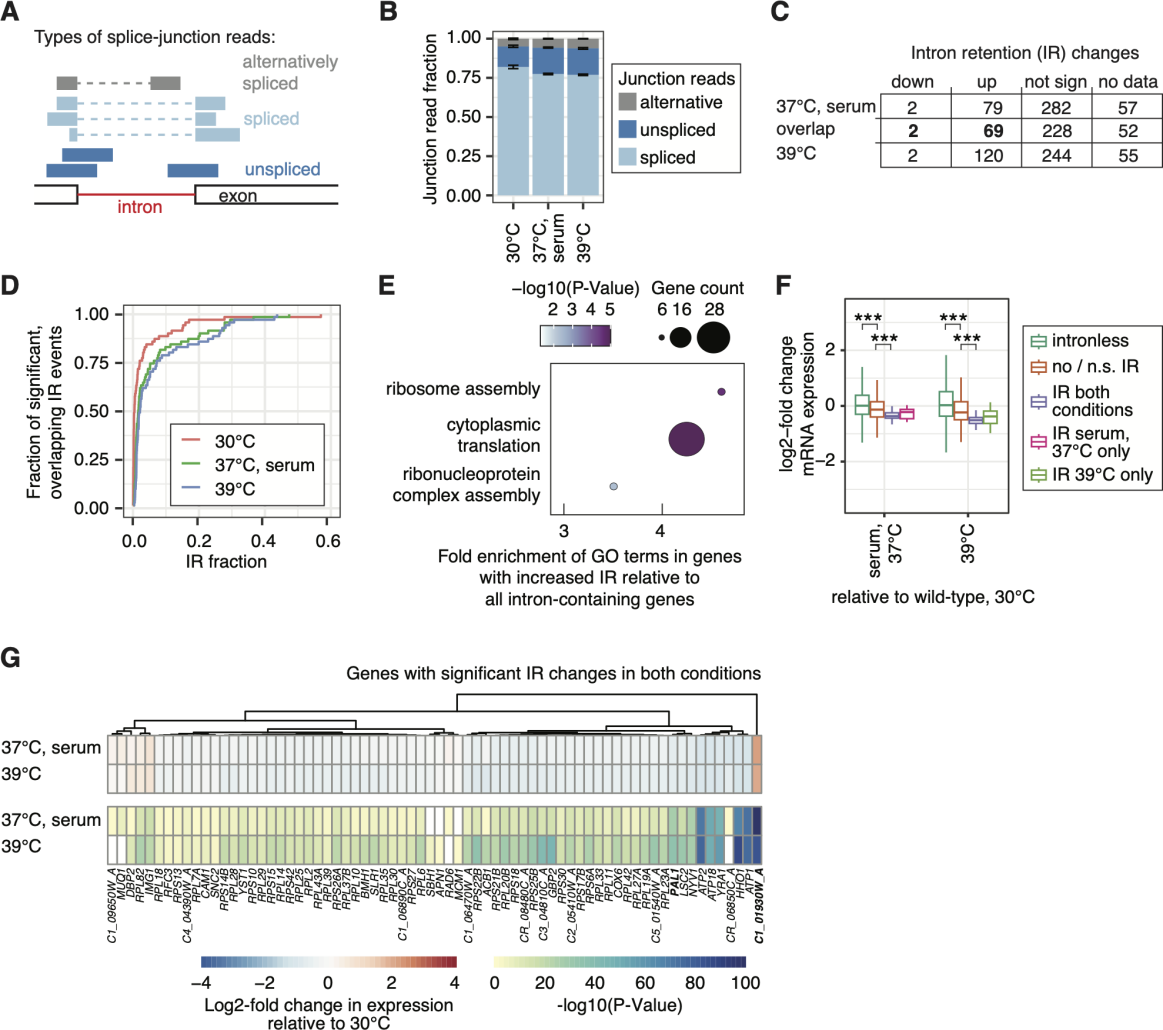
Intron retention is elevated in filaments. (**A**) Schematic of the three types of junction reads used to quantify pre-mRNA splicing. Spliced and unspliced reads are used to quantify intron retention. Alternatively spliced reads consider other forms of alternative splicing, such as alternative 5′ and 3′ splice site usage or exon skipping/inclusion that are joint in their counts for simplicity. (**B**) Increased fraction of unspliced reads in filament-inducing conditions. Error bars correspond to standard deviation across biological triplicates. (**C**) Table depicting the degree of IR across conditions. Introns were required to have at least 10 junction reads per replicate for quantification, and significance was assessed with the Student’s *t*-test (*P* < 0.05). Overlapping IR events are highlighted in bold, and IR value distribution is shown in panel D. (**D**) Cumulative distribution of the IR levels from significantly retained introns in both conditions. (**E**) Significant GO terms (Process) associated with intron-containing genes that showed retained introns in both filament-inducing conditions. (**F**) Log2-fold expression changes in filament-inducing conditions relative to 30°C for intronless and intron-containing genes. Intron-containing genes are grouped based on whether they were subject to significant differential IR during filamentation in both conditions, serum only, and 39°C only. Asterisks indicate significance in the Wilcoxon rank sum test: *P < 0.05 and ***P < 0.001. (**G**) Heatmap of log2-fold changes in gene expression relative to growth at 30°C, and heatmap of associated *P*-values for intron-containing genes that show significant IR in both conditions.

Next, we investigated the splicing profile of cells undergoing filamentation. Intron junctions for 420 introns extracted from the *Candida* Genome Database were used to quantify IR and other forms of alternative splicing, such as alternative 5′ and 3′ ends or exon skipping, by measuring spliced and unspliced reads (Fig. 5A). The increased fraction of unspliced reads in filaments suggests that IR is more prevalent in filaments compared to yeast and the most prevalent form of alternative splicing in these conditions (Fig. 5B). Approximately 87% of introns that showed a significant increase in IR (*P*-value > 0.05, median IR difference > 0) in serum filaments (69/79) were also retained at 39°C (Fig. 5C; Table S4). However, an additional 51 introns were significantly retained at 39°C, but not in serum (Fig. 5C; Table S4). Furthermore, among the overlapping 69 retained introns, there was a slightly higher degree of retention at 39°C than in serum (Fig. 5D; Table S4). Overall, this suggests that IR is upregulated in filaments, and filaments induced by growth at 39°C have higher levels of IR than filaments induced by serum. Among the intron-containing genes that showed a significant increase in IR in both filament-inducing conditions, there was a significant enrichment of genes involved in ribosome assembly and translation (Fig. 5E; Table S1). These enrichments suggest that IR during filamentation is non-random and biased toward genes encoding components of the translational machinery. Next, we wanted to investigate the possibility that IR serves as a mechanism to fine-tune gene expression. To do this, we assessed gene expression changes during filamentation for non-intron-containing genes and intron-containing genes that were or were not subject to a significant increase in IR during filamentation. Overall, we found that intron-containing genes tend to be downregulated compared to non-intron-containing genes (Fig. 5F). This pattern of downregulation for intron-containing genes was even more prevalent when considering genes that were subject to significant IR and was detectable for 90% of intron-containing genes with significant IR (Fig. 5G). Altogether, this suggests a possible role for IR in regulating gene expression changes during filamentous growth in both filament-inducing conditions.

### Substantial mRNA expression changes detected in the *prp19∆/∆* spliceosome mutant

**Fig 6:**
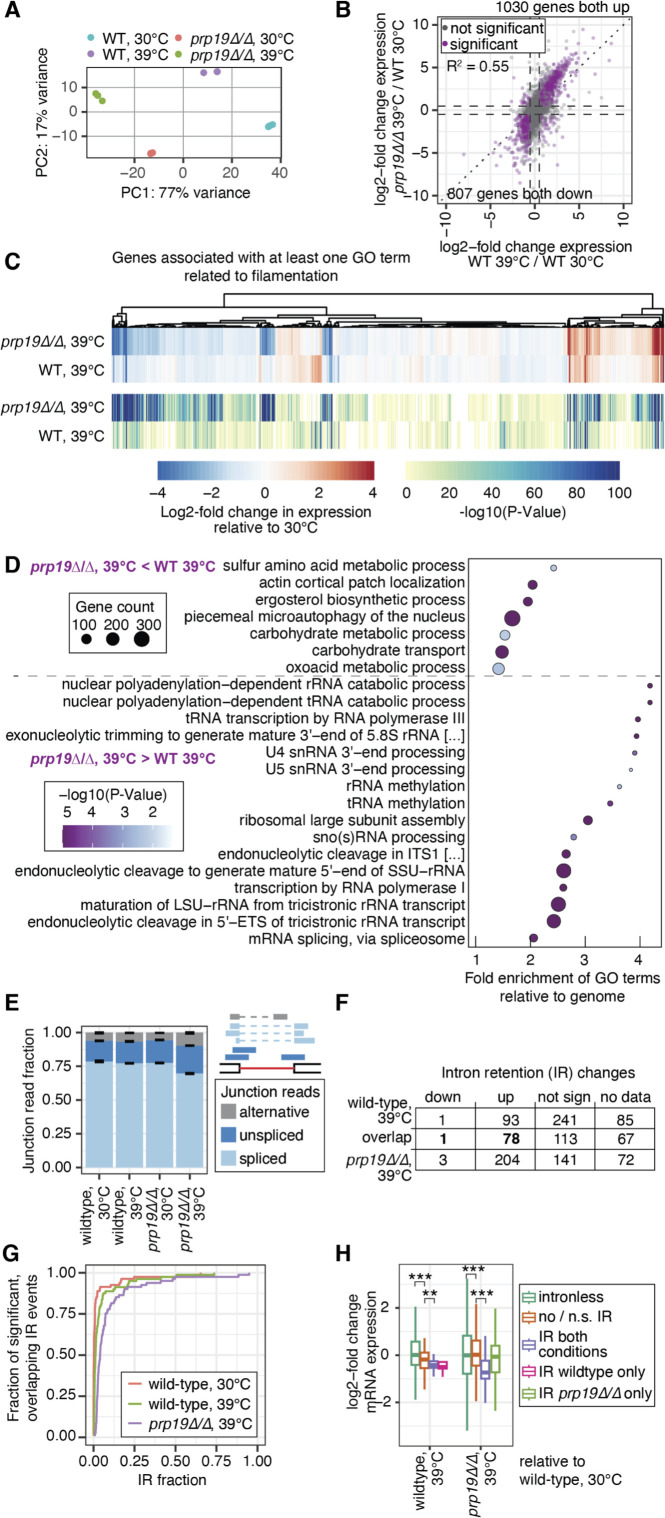
Intron retention is most pronounced in the *prp19∆/∆* mutant. (**A**) Principal component analysis for three RNA-seq replicates per condition. (**B**) Scatter plot of log2-fold expression changes between wild-type growth at 39°C and 30°C and the *prp19∆/∆* mutant at 39°C relative to wild-type at 30°C. Significant genes (shown in purple) have an absolute log2-fold change greater than 0.5 (dashed line) and adjusted *P*-value < 0.00001. The dotted line reflects the diagonal. Gene expression analysis was done with DEseq2. (**C**) Heatmap of log2-fold changes in gene expression relative to growth at 30°C, and heatmap of associated *P*-values for genes that are described by at least one GO term associated with filamentation. (**D**) Significant GO terms (Process, Bonferroni-corrected *P*-value < 0.01) associated with genes that are expressed with opposite signature in the *prp19∆/∆* mutant at 39°C (relative to wild type at 30°C) compared to gene expression differences between wild-type growth at 39°C relative to 30°C. (**E**) Strongly increased fraction of unspliced reads in the *prp19∆/∆* mutant at 39°C. Error bars correspond to standard deviation across biological triplicates. The schematic of reads refers to the same class of reads as in Fig. 5B. (**F**) Table depicting the degree of intron retention at 39°C for wild type and the *prp19∆/∆* mutant relative to wild type at 30°C. Introns were required to have at least 10 junction reads per replicate for quantification, and significance was assessed with the Student’s *t*-test (*P* < 0.05). Overlapping IR events are highlighted in bold, and IR value distribution is shown in panel G. (**G**) Cumulative distribution of the IR levels from significantly retained introns in both strains grown at 39°C. (**H**) Log2-fold expression changes in filament-inducing conditions relative to 30°C growth for intronless and intron-containing genes. Intron-containing genes are grouped based on whether they were subject to significant differential IR in both conditions, wild-type at 39°C only, and in the *prp19∆/∆* mutant at 39°C only. Asterisks indicate significance in the Wilcoxon rank sum test: **P < 0.01 and ***P < 0.001.

To gain further insight into how compromising spliceosomal genes disrupt filamentation, we conducted an additional RNA-Seq experiment aimed to identify which transcripts have impaired splicing in the *prp19∆/∆* mutant. For these experiments, wild-type (SN95) and *prp19∆/∆* cells were grown in biological triplicate for 4 hours in liquid YPD at 30°C or 39°C before harvesting cell pellets. Given that high temperature and serum-induced filaments gave similar splicing profiles (Fig. 5B), we focused here on growth at 39°C as the filament-inducing cue. RNA extraction, library preparation, and RNA-Seq data analysis were conducted as described above. Again, there was a strong similarity among biological replicates, and samples clustered based on growth condition and strain background (Fig. 6A). Interestingly, the expression profile of wild-type cells at 30°C was more similar to wild-type cells at 39°C than to the *prp19∆/∆* mutant at 30°C (Fig. 6A). We also investigated the gene expression changes of wild-type cells at 39°C and the *prp19∆/∆* mutant at 39°C in comparison to wild-type yeast at 30°C (Table S3). Out of 5,846 genes, 1,837 genes showed the same directionality (up or down) between the two genetic backgrounds, but the expression changes were mostly more extreme for the *prp19∆/∆* mutant (Fig. 6B). When specifically focusing on genes associated with filamentation, the *prp19∆/∆* mutant had a distinct expression profile at 39°C, whereby many expression changes were more pronounced or different than wild type (Fig. 6C), emphasizing that the filamentation response was perturbed. An extreme case was the expression of the hyphae-specific gene *ECE1*, which encodes for the peptide toxin Candidalysin. When comparing the wild-type strain at 30°C versus 39°C, this gene was induced the second strongest (335-fold); however, a smaller increase in expression (56-fold) was noted when comparing the *prp19∆/∆* strain at 39°C relative to wild type at 30°C.

Half of all genes were differentially expressed in the *prp19∆/∆* mutant compared to wild type at 39°C. GO analysis of up- and downregulated genes in the *prp19∆/∆* mutant compared to wild type at 39°C revealed expression signatures of altered processes that might lead to or contribute to the block in filamentation (Fig. 6D), e.g., the terms “actin cortical patch organization” and “ergosterol biosynthetic process” were enriched among the downregulated genes. Consistent with some degree of feedback regulation in this splicing gene mutant strain, genes associated with the GO term “mRNA splicing, via spliceosome” were enriched among upregulated genes. Taken together, this substantial alteration to the mRNA expression profile likely justifies the block in filamentation.

### Global intron retention is most pronounced in the *prp19∆/∆* spliceosome mutant

To grasp whether changes in pre-mRNA splicing are responsible for the vast transcriptome alteration, we analyzed the IR profile of the *prp19∆/∆* mutant. We reconfirmed our previous findings that filaments induced by growth at elevated temperatures have a greater level of global IR compared to yeast (Fig. 6E through G). We identified a significant increase in the fraction of unspliced reads in the *prp19∆/∆* mutant at 30°C and 39°C, suggesting a substantial level of IR in this mutant (Fig. 6E; Table S4). Interestingly, there was also a significant increase in the fraction of alternatively spliced reads in the *prp19∆/∆* mutant at 39°C (Fig. 6E; Fig. S2D). We also found alternative splicing to increase with temperature in the wild type (Fig. S2D). Approximately 84% of introns that showed a significant increase in IR in wild-type filaments (78/93) were also retained in the *prp19∆/∆* mutant at 39°C (Fig. 6F). However, an additional 126 introns were retained in *prp19∆/∆* at 39°C but not in wild-type filaments (Fig. 6F). Furthermore, introns were retained to a greater degree in the *prp19∆/∆* mutant at 39°C (Fig. 6G). IR and distinct gene expression changes to wild type were also detected at 30°C but to a lesser extent (Fig. S2A through C). Consistent with our previous analysis, intron-containing genes tend to be downregulated in their expression compared to non-intron-containing genes in wild-type filaments and even further downregulated when significant IR occurs at 39°C (Fig. 6H). However, in the *prp19∆/∆* mutant, there was no significant difference in gene expression between intron-containing and non-intron-containing genes (Fig. 6H; Fig. S2C). Genes that are subject to IR in both wild-type and the *prp19∆/∆* mutant at 39°C still tend to be downregulated in the mutant, however, this association is lost for genes subject to IR in the mutant only (Fig. 6H) and could reflect negative consequences for protein production as more of the expressed transcripts stay unspliced.

Overall, this analysis confirmed our finding that filaments induced by growth at elevated temperatures have a greater level of global IR than yeast grown at 30°C and revealed that deletion of the spliceosomal gene *PRP19* causes an even greater level of IR that could be associated with some of the observed expression changes relative to wild type at 39°C.

### Altered Tor1 signaling is not responsible for the inability of the *prp19∆/∆* spliceosome mutant to maintain filamentation

**Fig 7:**
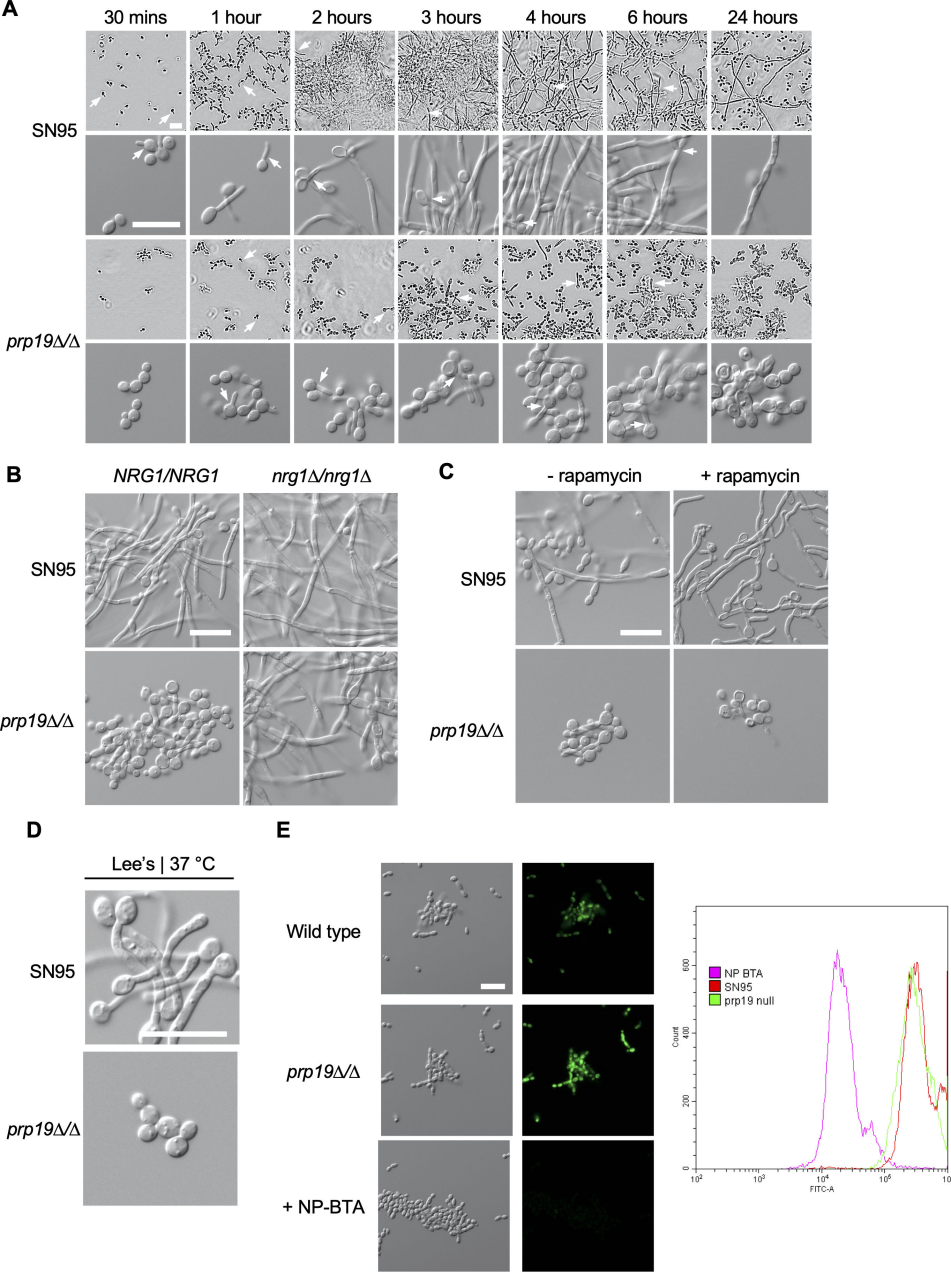
Loss of Prp19 blocks maintenance of filamentation but does not cause altered Tor1 activation. (**A**) Wild-type and *prp19∆/∆* cells were grown in YPD in static conditions for 24 hours at 39°C and imaged at the indicated time points. White arrowheads highlight examples of the initiation of polarized growth. Top images were taken using the IncuCyte S3 Live-Cell Analysis at 10× magnification. Bottom images were taken using Zeiss Axio Imager.M1 (Carl Zeiss) at 40× magnification. Scale bar is 20 µm. (**B**) Deletion of *NRG1* results in hyperfilamentation in wild-type and *prp19∆/∆* cells. Cells were grown for 4 hours in YPD in static conditions at 39°C. Scale bar is 20 µm. (**C**) Indicated cells were grown in the presence or absence of 20 µm rapamycin in YPD for 4 hours at 39°C before imaging. Scale bar is 20 µm. (**D**) Indicated cells were grown in Lee’s medium with shaking for 4 hours at 37°C before imaging. Scale bar is 20 µm. (**E**) Translation was quantified using a Click-iT protein synthesis kit. The indicated strains were grown for 4 hours at 30°C. Wild-type (SN95) cells were treated with 6.25 µM of the known translation inhibitor NP-BTA for 10 minutes. The I-homopropargylglycine alkyne methionine analog was added, cells were fixed, and the azide fluorophore was added before imaging on the GFP channel. Scale bar is 20 µm. Histograms depict relative fluorescence intensity (FITC-A) of a minimum of 20,000 events.

Given that the overall gene expression and IR profile of the *prp19∆/∆* mutant were significantly different from the wild type (Fig. 6), we next explored whether these changes triggered cellular signaling pathways that prevented filamentation. Interestingly, the *prp19∆/∆* mutant can initiate filamentation but fails to maintain polarized growth over time (Fig. 7A). Previous work in *C. albicans* found that maintenance of polarized growth required reduced Tor1 signaling in order to recruit the histone deacetylase Hda1 to promoters of filament-specific genes, resulting in chromatin changes that prevent the transcriptional repressor Nrg1 from binding (40). The ability of the *prp19∆/∆* mutant to initiate, but not maintain, filamentation along with transcriptional changes that pointed to altered Tor1 signaling (41) (Fig. S3A) led us to hypothesize that aberrantly high Tor1 signaling contributes to the block in filamentation. Furthermore, hyperfilamentation was observed upon *NRG1* deletion in the *prp19∆/∆* mutant, suggesting Nrg1 functions downstream of Prp19 and possibly the spliceosome (Fig. 7B). Tor1 signaling can be probed by visualizing phosphorylated Rps6 on a Western blot as Tor1 activation results in phosphorylation of this downstream effector. We did not detect higher levels of phosphorylated Rps6 in the *prp19∆/∆* mutant after 1 hour at 39°C, suggesting Tor1 activation is not elevated in this mutant (Fig. S3B). Even over a 4-hour time course at 39°C, higher levels of phosphorylated Rps6 were not detected in the *prp19∆/∆* mutant. Furthermore, conditions that block Tor1 signaling, including treatment with the Tor1 inhibitor rapamycin or growth in nutrient-limited Lee’s medium, did not support filamentation in the *prp19∆/∆* strain (Fig. 7C and D). Overall, these results suggest that the inability of the *prp19∆/∆* mutant to maintain filamentation is not due to upregulated Tor1 activation, and the ability of the *prp19∆/∆* mutant to filament in the absence of Nrg1 cannot be solely attributed to Tor1 signaling.

### Global protein translation is not affected by the deletion of Prp19

Our analysis revealed that genes involved in translation are subject to differential IR during filamentation (Fig. 5E). Therefore, we next explored whether the aberrant IR and gene expression profile of the *prp19∆/∆* mutant led to altered levels of protein synthesis that contributed to the block in filamentation. Efficient translation is important for filamentation, and small molecules that interfere with translation can block filamentation (42). To probe the levels of protein synthesis directly, we used a Click-iT protein synthesis assay kit, which uses an alkynylated methionine analog that is incorporated into newly translated proteins, allowing for the detection of a fluorescent signal upon a Click reaction with a green, fluorescent azide (37). While the translation inhibitor NP-BTA (37) resulted in a block in fluorescence as observed by microscopy and quantified using flow cytometry, no difference in fluorescence intensity was detected between the *prp19∆/∆* mutant and wild-type cells (Fig. 7E). Overall, this suggests that the expression changes observed for genes involved in translation, such as ribosomal protein genes (RPGs), do not result in an obvious global translational change in the *prp19∆/∆* mutant that could be responsible for the block in filamentation. Whether more subtle reductions in global translation levels occur upon deletion of *PRP19*, and whether this is responsible for the block in filamentous growth, remains to be explored.

## DISCUSSION

The ability to sense and respond to changes in the environment is critical for the opportunistic human fungal pathogen *C. albicans*. This is highlighted by *C. albicans’* ability to switch between yeast and filamentous growth states in response to diverse environmental cues, including elevated temperature. To uncover novel cellular processes that govern its dimorphic nature, we performed a functional genomic screen and identified 38 genes required for filamentation at 39°C. This revealed a novel role for mRNA splicing via the spliceosome in regulating filamentation.

A key question to emerge from our findings is why certain spliceosome components are required for filamentation, while others are dispensable. We found that approximately 45% (42/95) of genes encoding proteins with predicted roles in splicing are required for filamentation at 39°C, either in liquid or on solid YPD media. These included components that are essential or non-essential for cell viability. Upon closer examination of the genes for which transcriptional repression blocked filamentation, we noted that most spliceosome genes encoding proteins containing an Sm or Sm-like (Lsm) domain (12 out of 14 genes) are important for filamentation (Table 1). These RNA-binding proteins form heteroheptameric Sm or Lsm rings, which interact with spliceosomal snRNAs to assemble the structural core of the snRNPs. In *Arabidopsis,* the Lsm spliceosome subcomplex differentially accumulates in response to cold and salt stress to differentially regulate the efficiency and accuracy of splicing of specific introns, depending on the environmental context (43). Ultimately, the complex ensures that in response to these abiotic stressors, there are appropriate levels of functional transcripts, and likely proteins, to adapt to the changing environment (43). Our finding that *C. albicans* cannot respond to the temperature upshift and undergo filamentation when the Lsm complex is disrupted is in line with the Lsm complex having an environmentally responsive role in splicing. Furthermore, the Lsm complex has additional roles in mRNA decay, and the Sm complex has additional roles in binding telomerase RNA in eukaryotes (44–46). Hence, it is possible that other RNA metabolic processes are also important for filamentation. Another explanation as to why only some spliceosome components are required for filamentation could be due to transcript specificity. In *S. cerevisiae,* microarray profiling of 18 mutants of different core spliceosome components revealed clear transcript-specific splicing defects (47). Transcript-specific effects have also been observed using RNA interference to knockdown splicing regulators in *Drosophila* (48). This indicates that the spliceosome can differentiate among pre-mRNA substrates to regulate their splicing efficiency (47, 48). Given that transcripts are differentially affected by particular spliceosome mutations, it is possible that those components whose depletion does not block filamentation are less important for the adequate splicing of key transcripts required for this developmental transition. In the future, it will be interesting to characterize the splicing profile of such filament-competent spliceosome mutants. Finally, it is possible that for at least some of the 42 proteins, the complexes to which they belong are less able to function at high temperatures when a constituent is depleted from the cell. Future work to assess subcomplex stability over a temperature gradient will be required to further explore this possibility.

We observed that the level of IR in filaments was significantly higher than in yeast and accompanied by reduced expression of these genes, e.g., involved in ribosome biogenesis (Fig. 5). Paradoxically, the majority of ribosome biogenesis genes are, however, increased in their expression upon filamentation (Fig. 4C) and intronless, which suggests a separate mode of expression regulation based on their gene architecture for these two subsets of genes. Interestingly, a positive correlation between IR and ribosome biogenesis was observed during meiosis in *S. cerevisiae* (49). In both *C. albicans* and *S. cerevisiae*, RPGs are the largest functional class of intron-containing genes and thus comprise most of the splicing substrates in the cell (50). During early meiosis, nutrient signaling causes repression of RPG expression, which reduces competition for the splicing machinery and leads to increased global splicing efficiency in *S. cerevisiae* (49). However, in late meiosis when RPG expression is reactivated, there is again competition for the spliceosome that causes splicing efficiency to go down (49). Interestingly, reducing RPG transcription through genetic means or by rapamycin treatment improves splicing efficiency and rescues the splicing and temperature-sensitive growth defects of *prp4* and *prp11* spliceosome mutant strains in *S. cerevisiae* (49). In our study, there are several possible explanations as to why rapamycin treatment did not restore filamentation in the *prp19∆/∆* mutant. For one, we did not perform RNA-Seq on rapamycin-treated cells, and it is therefore unknown whether compound treatment sufficiently repressed RPG expression to a level whereby splicing of other transcripts was improved. Furthermore, given what we know about transcript specificity, it is possible that even if overall splicing efficiency was improved, key transcript(s) are not efficiently spliced in the absence of Prp19. Additionally, it is established that rapamycin inhibits filamentation on some types of agar media and, therefore, could be having off-target effects that prevent it from restoring filamentation due to increased splicing efficiency (51). In the future, it will be interesting to better understand the cause-and-effect relationship of IR and RPG expression. One hypothesis is that in the high-temperature environment, a reduction in splicing efficiency leads to a compensatory upregulation of translational machinery to deal with suboptimal substrates. Alternatively, ribosomes may be upregulated in filaments to synthesize proteins needed to drive the dramatic change in cell morphology, and as a result, the increase in RPG expression overwhelms the spliceosome and causes the high level of IR observed. Further dissecting these models will help us better understand the complexity of this relationship and why many spliceosome mutants are unable to undergo filamentation.

Future research should focus on elucidating the biological function of IR during filamentation. In wild-type filaments, genes with introns were expressed at lower levels. Therefore, it is plausible that during filamentation, increased IR is a means to fine-tune gene expression in response to a changing environment. In the *prp19∆/∆* mutant, the number of retained introns and the degree to which they were retained were significantly increased, but the association between intron-containing genes having decreased expression was lost. Furthermore, gene expression, including those with filament-related functions, was dysregulated. Therefore, it is possible that due to aberrant IR in this mutant and gene expression changes, signaling required for filamentation is lost and so filamentation cannot occur. Given that genes involved in translation were subject to increased IR in wild-type filaments, protein translation could also be at play. Stop codons are typical in yeast and other eukaryotic introns, and thus, IR can prevent translation and lead to altered protein levels (52). In a study that matched antibody-based profiling with RNA-Seq samples for nine human tissue types, IR was significantly associated with lower protein levels, and intron-retaining transcripts that evaded nonsense-mediated decay were not actively translated (53). In *S. cerevisiae*, IR induced by lithium chloride treatment was correlated with a decrease in the respective protein level (25). This possibility that IR is a means to control protein expression is interesting considering evidence that many genes that are transcriptionally induced during *C. albicans* morphogenesis show significantly reduced translational efficiency (54). Though on a gross cellular level, translation was not altered in *prp19∆/∆*, it is possible that levels of specific proteins important for filamentation were altered by increased IR. Future work should take a global proteomic approach to determine how the translatome is affected by spliceosome mutation.

The importance of the spliceosome in *C. albicans* filamentation adds to the growing body of literature that splicing is important for virulence in diverse fungi (31, 55). For *Cryptococcus neoformans,* in which the majority of genes possess introns, IR fine-tunes gene expression levels in response to growth conditions, suggesting it could provide a mechanism to promote pathogen survival under diverse environmental cues (56). Prior to this work, only one splicing-related factor, the SR-like protein Slr1, had been shown to play a role in regulating hyphal growth on serum agar plates and be important for virulence during intravenous mouse infection (57). In *Aspergillus nidulans*, the deletion of SwoK, an SR-like protein involved in splicing, results in cells that can germinate but germ tubes fail to maintain polarity (58). For *Fusarium graminearum*, the causative agent of head blight in wheat, cells lacking the spliceosome component Srp1 are non-pathogenic in wheat head and corn silk infections (59). Finally, in *Ophiostoma novo-ulmi*, the causative agent of Dutch elm disease, deletion of Col1, a U4/U6 splicing factor, dramatically reduces filamentous mycelial growth (60). Overall, these data suggest that targeting the spliceosome may be a promising avenue for the development of broad-spectrum anti-virulence antifungals. However, due to the highly conserved nature of the spliceosome, further exploration into targets that can be inhibited in a fungal-selective manner must be prioritized.

## MATERIALS AND METHODS

### Strains and culture conditions

Details regarding all oligonucleotides, strains, and plasmids used in this study can be found in Table S5 and Table S6. All *C. albicans* strains were archived in YPD medium (1% yeast extract, 2% peptone, and 2% glucose) supplemented with 25% glycerol at −80°C. YPD medium was used as the culture medium unless otherwise specified. 2% agar was added for solid growth conditions. To transcriptionally repress target gene expression driven by the *tetO* promoter in GRACE strains, 0.05–20 µg/mL doxycycline (catalog no. DB0889; BioBasic; dissolved in water) was added to the medium.

### Liquid-arrayed screen for regulators of morphogenesis

Arrayed strains were inoculated into 96-well flat-bottom microtiter plates, each containing 100 µL of YPD medium, and allowed to grow overnight at 30°C in static conditions. The following day, approximately 0.5 µL of the overnight cultures was transferred using a 96-well pinner into fresh YPD medium containing 0.05 µg/mL DOX and grown overnight at 30°C. The next day, the same procedure was used to transfer cells into two identical 96-well plates, each containing fresh YPD media supplemented with 0.05 µg/mL DOX. One plate was incubated at 39°C in the IncuCyte Live Cell Imager, which took images every hour for 24 hours with a 20× magnification lens. The other plate was incubated at 30°C, and OD_600_ was measured at 24 hours using a spectrophotometer (Molecular Devices). The OD_600_ of each strain at 24 hours was normalized to the OD_600_ of a wild-type control (CaSS1) for each batch to calculate what percentage of wild-type growth each strain achieved. Strains that grew less than 25% relative to the wild type were considered to have a severe growth defect and were not prioritized. The images taken at 4 hours were used to score each strain based on their degree of filamentation on a scale of 0–3. A score of 0 indicates a complete block in filamentation, a score of 1 indicates short filaments or a majority yeast population with few filaments, a score of 2 indicates majority filaments with a few yeast, and a score of 3 indicates a wild-type level of filamentation. Strains that did not proliferate based on analyzing images at 4 and 24 hours at 39°C were removed. Strains that were scored as 0 or 1 at 4 hours were also analyzed using the 12-hour images to ensure filamentation was not delayed. Any strains that were considered hits were shown to be capable of filamenting in the absence of DOX.

### Filamentation assays

For high temperature-induced filamentation, cells were incubated in YPD medium at 39°C. For serum-induced filamentation, the YPD medium was supplemented with 10% heat-inactivated newborn calf serum (catalog no. 26010074; Gibco), and cultures were incubated at 37°C without shaking. For nutrient deprivation-induced filamentation, Lee’s medium was prepared according to previously established protocols (10) and cells were incubated at 37°C. The timing (4–6 hours) and whether the incubation was done in static or shaking conditions are indicated in the text and figure legends. For arrayed analysis of filamentation on solid, approximately 1 µL of the overnight culture was transferred onto the respective agar media using a plastic pinner and incubated for 72 hours at 37°C for serum-induced filaments and 39°C for high temperature-induced filaments. For the analysis of filamentation on solid highlighted in Fig. 2, 5 µL of the overnight culture was spotted onto the respective agar media using a micropipette. Cell morphology in liquid was visualized using differential interference contrast (DIC) microscopy on a Zeiss Axio Imager.M1 (Carl Zeiss) at 40× magnification or using the IncuCyte Live Cell Analysis System at 20× magnification. Colony morphology on solid media was captured using a Bio-Rad ChemiDoc imaging system. All presented images are representative of two biological replicates.

### Testing for essentiality

GRACE strains were grown overnight in YPD in 96-well plates at 30°C. The following day, cells were pinned into fresh YPD-containing 96-well plates, with and without the addition of 100 µg/mL DOX and grown overnight at 30°C. The next day, a metal replicator was used to transfer approximately 2 µL of each culture onto YNB agar plates, with and without 100 µg/mL DOX, and these plates were grown for 2 days at 30°C. The plates were imaged, and each spot was scored for essentiality on a scale of 0–4, where a score of 0–2 indicates a non-essential gene and a score of 3 or 4 indicates an essential gene (37). Scores in the absence of DOX were used to ensure strains could grow robustly in the absence of DOX. Two biological replicates were conducted.

### Spliceosome component identification

To identify *C. albicans* genes with a role in mRNA splicing via the spliceosome, all genes annotated to this GO process term were downloaded from the CGD website (26). Additionally, all genes annotated to this GO process term were downloaded from the *Saccharomyces* Genome Database (36) and transformed to their *C. albicans* homolog using FungiDB (61). These two lists were cross-referenced to ensure all homologs were covered, and manual curation was performed to remove any spurious genes.

### Quantitative reverse transcription-PCR

GRACE mutants and the parent strain (CaSS1) were grown overnight in YPD, and the following day, they were sub-cultured to an approximate OD_600_ of 0.1 in YPD containing 0.05 µg/mL DOX (essential genes) or 20 µg/mL DOX (non-essential genes) in the absence of DOX. All cultures were grown overnight at 30°C with shaking. The following day, the overnight cultures were sub-cultured into the same DOX conditions and grown at 30°C with shaking until the mid-log phase when pellets were collected, flash frozen, and stored at −80°C. Quantitative reverse transcription-PCR was performed as previously described (37). Briefly, cells were lysed by bead beating, followed by RNA extraction using the RNAeasy kit (Qiagen) and DNase treatment using the DNA-free DNA removal kit (Invitrogen). The iScript cDNA synthesis kit (Bio-Rad) was used for cDNA synthesis. The kit includes a mix of oligo-dT and random hexamers for priming the cDNA synthesis. The Fast SYBR green master mix (Applied Biosystems) was used for PCR with the Bio-Rad CFX-384 real-time system. The PCR cycling conditions consisted of an initial denaturation at 95°C for 3 minutes, followed by 40 cycles of denaturation at 95°C for 10 seconds and annealing/extension at 60°C for 30 seconds. All reactions were conducted in technical triplicate for two separate biological replicates. Data analysis was carried out using the Bio-Rad CFX Manager 3.1 software. All data were normalized to the reference genes, *ACT1* and *GPD1*. Error bars represent the standard error of the mean of technical triplicate measurements.

### *ACT1* splicing assay

To test for *ACT1* splicing, reverse transcription-PCR was performed on RNA extracted from wild-type and *prp19∆/∆ C. albicans*. Overnight cultures were grown in YPD at 30°C with shaking, and the next day, they were sub-cultured to an approximate OD_600_ of 0.1 and grown for 4 hours at 30°C with shaking. The same procedures for RNA extraction, DNase treatment, and cDNA synthesis were followed as described above for the quantitative reverse transcription-PCR. A volume of 1 µL of the cDNA product was then mixed with 0.1 µL of each *ACT1* primer at 100 µM (oLC9781 and oLC10309), 2.5 µL of the supplied 10× reaction buffer, 2 µL of the supplied 2.5 mM dNTP mix, 0.25 µL of TAKARA Taq DNA polymerase, and 19 µL of dd H_2_O and subjected to a 30-cycle PCR reaction of 94°C for 30 seconds, 52°C for 45 seconds, and 72°C for 60 seconds. PCR products were run on a 2% agarose gel at 100 V for 60 minutes, and the SYBR Safe-stained gels were imaged on a ChemiDoc.

### Cell growth and RNA preparation for RNA sequencing

To prepare cell pellets for RNA extraction, saturated overnight cultures were sub-cultured to an approximate OD_600_ of 0.1 and grown for 4 hours in the indicated condition, with shaking. The same procedures for RNA extraction and DNase treatment were followed as described above for the quantitative reverse transcription-PCR. Sample libraries were prepared using the Illumina Stranded mRNA Ligation Kit and sequenced with the Illumina Novaseq 6000 using a 100 bp paired-end protocol and multiplexing to obtain >25 million paired-end reads/sample.

### RNA-Seq data processing and mapping

Paired-end RNA-seq data were trimmed with cutadapt/2.10 to remove any residual adaptor sequences using the following flags and settings: cutadapt --cores=0 -b CTGTCTCTTATACACATCT -B CTGTCTCTTATACACATCT -e 0.1 -O 10 -m 16 --output=R1t.fastq.gz --paired-output=R2t.fastq.gz $R1 $R2 (62). FastQC (FastQC/0.11.9; http://hannonlab.cshl.edu/fastx toolkit/index.html) analysis was performed on untrimmed and trimmed data. Trimmed data were mapped to the *Candida albicans* SC5314 genome, version A22 with HISAT version 2.2.1 using the following command: hisat2 --summary-file summary.txt --max-intronlen 2000 -x C_albicans_SC5314_A22 --rna-strandness FR −1 $R1 −2 $R2 -S out.sam --novel-splicesite-outfile novelSS.txt (63). Using samtools, bedtools, and igvtools (SAMtools/1.16.1, BEDTools/2.30.0, igvtools 2.14.1), hisat2 output was further filtered to retain only mapped reads and converted into bam- and coverage-files (tdf and bedgraph) for visualization in IGV and downstream analyses (64–66). All further analyses on differential expression and pre-mRNA splicing were done for A and B alleles, although all visualizations consider only A allele data.

### Differential expression analysis and visualization

Reads per gene were counted for differential expression analysis with a custom bash script. In brief, mapped reads were split into forward and reverse reads according to their orientation in the pair with samtools view -h -b -f 0 × 40/ f 0 × 80. With bedtools intersect, stranded overlap to annotated genes in *C. albicans* was achieved, and reads per gene were counted using awk and bash commands. Introns [including 5′ untranslated region (UTR) introns downloaded from CGD] and approximately annotated UTRs from references (27, 67) were included in the gene annotation. Gene expression was quantified and analyzed with custom R scripts. For differential expression (DE) analysis, the DESeqDataSetFromMatrix, DESeq, and results functions from the DESeq2 package in Bioconductor were used (68). Principal component analysis was carried out using the vst and plotPCA functions of the DESeq2 package. Ggplot2 and functions within the package were used for other visualizations. To plot DE changes as heatmaps, the pheatmap package was used. In general, an adjusted *P*-value of 0.00001 and an absolute log2-fold change of 0.5 were used as cutoff to call genes up- or downregulated in comparisons between two conditions, e.g., filament-inducing condition (wild-type growth at 39°C or wild-type growth in serum at 37°C) relative to wild-type yeast growth at 30°C.

### Intron retention quantification

For pre-mRNA splicing quantification, annotated introns within the *Candida* Genome Database were considered (26 5′UTR- and 396 CDS introns, Table S4). IR was quantified and analyzed with custom bash and R scripts. Mapped reads were split into forward and reverse reads according to their orientation in the pair with samtools view -h -b -f 0 × 40/ f 0 × 80. With bedtools intersec, stranded overlap to introns in *C. albicans* was achieved. An overlap of at least three bases was required to consider reads to be overlapping with the intron. Read coordinates formatted as a bed-file were used to classify reads according to their splicing status. Split reads (block count in bed-file > 1) were either classified as “spliced” or “alternatively spliced” (Fig. 5A). “Spliced” reads had split junctions matching the precise start and end of an annotated intron. “Alternatively spliced” reads deviated from this but showed overlap with the intron. Continuous reads (block count in bed-file = 1) are either “unspliced” junction reads or “intronic” reads. If the start and end of the read were located within the intron coordinates, a read was defined as “intronic” and otherwise “unspliced.” Once classified reads within the different categories were counted across all introns together (Fig. 5B and 6E) or counted for each intron separately. IR for each intron was quantified as IR fraction, being the fraction of “unspliced” reads over the sum of “unspliced” and 2× “spliced” junction reads. Factor 2 for “spliced” junction reads was introduced to account for the fact that one unspliced transcript can give rise to two “unspliced” reads, but a spliced transcript can only yield one “spliced” read. For further analysis, a minimum junction read count of 10 was required per intron. Two or more replicates per condition were required to pass the cutoff to assess the significance in IR differences. The Student’s *t*-test was performed to assess the significance in IR differences. A *P*-value smaller than 0.05 was used as a significance cutoff for downstream analysis and classification.

### Gene Ontology analysis

GO term enrichment was performed using the combined GO annotations from *Candida* genome database ( gene_association.cgd.gz, from 08 October 2023, gaf-version: 2) with the most recent version from 17 January 2024 (https://geneontology.org/, gaf-version: 2.2) and the GO terms from *Saccharomyces cerevisiae* orthologs (https://geneontology.org/, gaf-version: 2.2, 18 January 2024). *S. cerevisiae* orthologous genes were matched according to the information on CGD. GO term annotations were extended to all parents of individual GO terms using the “get_parent_nodes” function from the GOfuncR package. Occurrences of GO terms were counted in each target gene group and compared to their frequency in the gene background. A total of 38 genes that were identified as important for filamentation at 39°C formed the target gene group for the functional genomics screen (Fig. 1C). The respective background gene set corresponds to all genes within the GRACE library. For GO term enrichment among groups of differentially expressed genes, we distinguished between upregulated genes (log2-fold change of differential expression > 0.5 and adjusted *P*-value < 0.00001) and downregulated genes (log2-fold change of differential expression > 0.5 and adjusted *P*-value < 0.00001). In this case, the gene background was formed by all genes that were quantified as expressed in the RNA-seq data. Gene background for GO term enrichment analysis in genes with IR (*P* < 0.05, difference in IR > 0 to wild type at 30°C) was all intron-containing genes. To assess the significance of GO term enrichment, we performed the Fisher’s exact test and multiple testing correction (Bonferroni correction). A *P*-value cutoff of <0.01 was used to call positively enriched GO terms for “Biological Process.” Visualization of the GO terms for “Biological Process” was always done for the most specific and significantly enriched terms, i.e., terms that did not have any children that were also significantly enriched (except for Fig. 1C). For GO terms associated with filamentation, the following GO term IDs were used: 0030447, 0036180, 0036178, 0036170, 0030446, 0030448, 0036177, 2000221, 0000902, 0009272, 0010570, 0009277, 0001411, 0036171, and 0036267.

### Computational resources and scripts

Data processing, mapping, and IR quantification were done on the high-performance computing system at FU Berlin (69). Most analysis using R was done within the framework of RStudio. R, bash, and job scripts can be found on Github at https://github.com/lherzel/CaFilamentationRNAseq.

### Western blotting

Wild-type and *prp19∆/∆* strains were grown overnight in YPD at 30°C with shaking. The next day, strains were sub-cultured to an approximate OD_600_ of 0.2 in 100 mL YPD (*prp19∆/∆*) or 50 mL YPD (SN95) and grown at 39°C with shaking for 1 hour. Cell pellets were collected by centrifugation, washed 1× in ice-cold water, then flash frozen with liquid nitrogen and stored at –80°C. Pellets were resuspended in 650 µL of buffer containing 427 mM NaOH and 1.73% beta-mercaptoethanol in water, then incubated on ice for 5 minutes before 150 µL of 50% TCA was added and mixed by tube inversion and another 5-minute incubation on ice. The protein pellet was collected by centrifugation and then resuspended in loading buffer (80 mM TrisHCl, pH 6.8, 10% SDS, 200 mM NaEDTA, and 0.2 mg/mL bromophenol blue) and incubated at 42°C for 10 minutes. Cell debris was collected and removed by centrifugation. To approximate protein concentration, 7 µL of each sample was loaded onto a resolving gel containing 2,2,2-trichloro-ethanol at a final concentration of 0.5% (vol/vol) and imaged on a ChemiDoc. A volume of 20 µL of each sample diluted in loading buffer containing the same amount of protein was loaded on precast Novex 10% Tris Glycine Mini Gels (Fisher Scientific) to separate proteins. Separated proteins were transferred to polyvinylidene difluoride membranes at 300 mA for 60 minutes. Blots were blocked with 3% bovine serum albumin (BSA). Phospho-Rps6 was detected using anti-phospho (S/T) Akt substrate rabbit polyclonal antibody (Cell Signaling Technology, catalog #9611). Total Rps6 was detected using an anti-S6 polyclonal antibody (R&D, catalog #AF5436). Blots were washed with TBS-T and incubated with HRP-conjugated secondary antibody diluted 1:5,000 in block solution. Blots were washed again with TBS-T before signal detection with Clarity Western ECL substrate. The blots were performed in biological duplicate.

### Translation assay

The translation assay was conducted using the Click-iT HPG Alexa Fluor 488 Protein Synthesis Assay Kit (Thermo Fisher C10428), with slight modifications to the manufacturer’s protocol. Wild-type and *prp19∆/∆* overnight cultures in YPD were sub-cultured into a synthetic defined medium, which lacked amino acids and ammonium sulfate but contained 2% glucose, histidine, and arginine. The cells were grown at 30°C with shaking until they reached the mid-log phase. For the positive control, a 500 µL aliquot of wild-type cells was treated with 6.25 µM NP-BTA for 10 minutes under shaking conditions at 30°C. Following this, the L-homopropargylglycine alkyne methionine analog was added to each culture at a 1:1,000 dilution and incubated under shaking conditions at 30°C for 30 minutes. Subsequently, the cells were washed with 1× PBS, and the cells were fixed by adding an equal volume of 70% ethanol. The mixture was gently rocked for 1 hour at room temperature before washing twice with 3% BSA. The resulting cell pellets were resuspended in a 500 µL reaction cocktail containing the azide fluorophore, prepared following the manufacturer’s protocol. The suspension was incubated for 30 minutes with gentle shaking at room temperature in the dark. Following incubation, the samples were washed once with the kit’s rinse buffer, pelleted, and then resuspended in 30 µL of 1× PBS for subsequent imaging. Cells were imaged using both DIC microscopy and the EGFP channel on a Zeiss Axio Imager.MI, with the same exposure time used for all samples. To quantify the fluorescence of each sample, a CytoFlex Flow Cytometer (Beckman Coulter) was used. The cell suspensions, in approximately 200 µL of 1× PBS, were added to a flat-bottom, transparent, 96-well plate (Beckman Coulter). Each sample was analyzed using the CytExpert Software until approximately 20,000 events were recorded. Appropriate gating strategies were applied to exclude debris and doublets. The histograms represent the fluorescein isothiocyanate (FITC) values for each event within the population after gating. This experiment was conducted in biological duplicate with the same results.

### Strain construction

GRACE strains that were not available in published collections were constructed as described previously (37). GRACE strains generated for this study are listed in Table S6.

#### CaLC7774

Both alleles of *PRP19* were deleted using a transient CRISPR approach (70). The nourseothricin (NAT) deletion cassette with homology to *PRP19* was PCR amplified from pLC49 using oLC9705 and oLC9706. oLC6924 and oLC6925 were used to amplify Cas9 from pLC963. Two fragments for the single guide RNA (sgRNA) were amplified from pLC963 with oLC5978 and oLC9704 (fragment A) and oLC5980 and oLC9703 (fragment B). Fusion PCR with nested primers oLC5981 and oLC5979 was performed on fragments A and B to produce the sgRNA. All three PCR products were transformed into SN95. NAT-resistant transformants were selected on YPD plates supplemented with 150 µg/mL NAT. NAT-resistant colonies were PCR tested for upstream integration of the NAT cassette with oLC275 and oLC9707 and downstream integration of the NAT cassette with oLC274 and oLC9709. Homozygous deletion of *PRP19* was confirmed by the lack of amplification of a wild-type allele using oLC9707 and oLC9708. Expression of the FLP recombinase was induced by growing this colony in YNB-BSA (0.17% nitrogen base, 0.4% bovine serum albumin, 0.2% yeast extract, and 2% maltose) to excise the NAT marker.

#### CaLC8643

The *ACT1* intron was deleted using a transient CRISPR approach (70). Two fragments for the sgRNA were amplified from pLC963 with oLC5978 and oLC10k751 (fragment A) as well as oLC5980 and oLC10k752 (fragment B). Fusion PCR with nested primers oLC5981 and oLC5979 was performed on fragments A and B to produce the sgRNA targeting the intron. oLC6924 and oLC6925 were used to amplify Cas9 from pLC963. The repair cassette was amplified from pLC620 using oLC10k753 and oLC10k754. The forward primer has homology to the *ACT1* 5′ UTR and pLC620 promoter. The reverse primer has homology to the end of the *ACT1* promoter and the *ACT1* ORF flanking the intron (base pairs 1–10 and 669–728). All three components were transformed into CaLC239. NAT-resistant colonies were PCR tested with oLC9781 and oLC10k309, which flank the *ACT1* intron. Amplification of a 207 base-pair piece and lack of amplification of an 865 base-pair piece confirmed the intron was deleted from both alleles. The NAT resistance marker was flipped out by growing a single transformation colony in YNB supplemented with 1% BSA for 24 hours. The absence of an intron-containing *ACT1* transcript was further confirmed by performing reverse transcriptase-PCR on this strain and visualization of the products on a gel, confirming the lack of a larger intron-containing amplicon.

#### CaLC8644

The *ACT1* intron was deleted using a transient CRISPR approach (70). Two fragments for the sgRNA were amplified from pLC963 with oLC5978 and oLC10k751 (fragment A) as well as oLC5980 and oLC10k752 (fragment B). Fusion PCR with nested primers oLC5981 and oLC5979 was performed on fragments A and B to produce the sgRNA targeting the intron. oLC6924 and oLC6925 were used to amplify Cas9 from pLC963. The repair cassette was amplified from pLC620 using oLC10k753 and oLC10k754. The forward primer has homology to the *ACT1* 5′ UTR and pLC620 promoter. The reverse primer has homology to the end of the *ACT1* promoter and the *ACT1* ORF flanking the intron (base pairs 1–10 and 669–728). All three components were transformed into CaLC7774. NAT-resistant colonies were PCR tested with oLC9781 and oLC10k309, which flank the *ACT1* intron. Amplification of a 207 base-pair piece and lack of amplification of an 865 base-pair piece confirmed the intron was deleted from both alleles. The NAT resistance marker was flipped out by growing a single transformation colony in YNB supplemented with 1% BSA for 24 hours. Absence of an intron containing *ACT1* transcript was further confirmed by performing reverse transcriptase-PCR on this strain and visualization of the products on a gel, confirming the lack of a larger intron-containing amplicon.

#### CaLC8555

Both alleles of *NRG1* were deleted using a transient CRISPR approach (70). The NAT deletion cassette with homology to *NRG1* was PCR amplified from pLC49 using oLC6663 and oLC6664. oLC6924 and oLC6925 were used to amplify Cas9 from pLC963. The sgRNA construct was amplified from pLC1075 with oLC5979 and oLC5981. All three PCR products were transformed into CaLC7774. NAT-resistant transformants were selected on YPD plates supplemented with 150 µg/mL NAT. NAT-resistant colonies were PCR tested for upstream integration of the NAT cassette with oLC275 and oLC6669 and downstream integration of the NAT cassette with oLC274 and oLC6670. Homozygous deletion of *NRG1* was confirmed by the lack of amplification of a wild-type allele using oLC4106 and oLC4107.

## Data Availability

The sequencing data from this study have been submitted to the NCBI Gene Expression Omnibus (GEO; http://www.ncbi.nlm.nih.gov/geo/) under accession number GSE262764. R, bash, and job scripts can be found on Github at https://github.com/lherzel/CaFilamentationRNAseq.

## SUPPLEMENTAL MATERIAL

Fig. S1 mbio.01535-24-s0001.tiff Characterization of GRACE strains from phenotypic screen.

Fig. S2 mbio.01535-24-s0002.tiff Characterization of gene expression and splicing changes at elevated temperature and in the *prp19*∆/∆ mutant.

Fig. S3 mbio.01535-24-s0003.pdf Gene expression downstream of the TOR signaling cascade is altered in the *prp19*∆/∆ mutant, but Tor1 activation is not upregulated.

Table S1 mbio.01535-24-s0004.xlsx GO term enrichments.

Table S2 mbio.01535-24-s0005.xlsx GRACE strain morphology, growth, and essentiality scores.

Table S3 mbio.01535-24-s0006.xlsx Differential expression analysis.

Table S4 mbio.01535-24-s0007.xlsx Intron retention quantification.

Table S5 mbio.01535-24-s0008.xlsx Oligonucleotides used in this study.

Table S6 mbio.01535-24-s0009.docx Strains and plasmids used in this study.

ASM does not own the copyrights to Supplemental Material that may be linked to, or accessed through, an article. The authors have granted ASM a non-exclusive, world-wide license to publish the Supplemental Material files. Please contact the corresponding author directly for reuse.
