# Supplementary figures and images for: The spliceosome impacts morphogenesis in the human fungal pathogen Candida albicans
Source: mBio. 2024 Jul 9;15(8):e01535-24. doi: 10.1128/mbio.01535-24 (PMC11323467; doi:10.1128/mbio.01535-24)

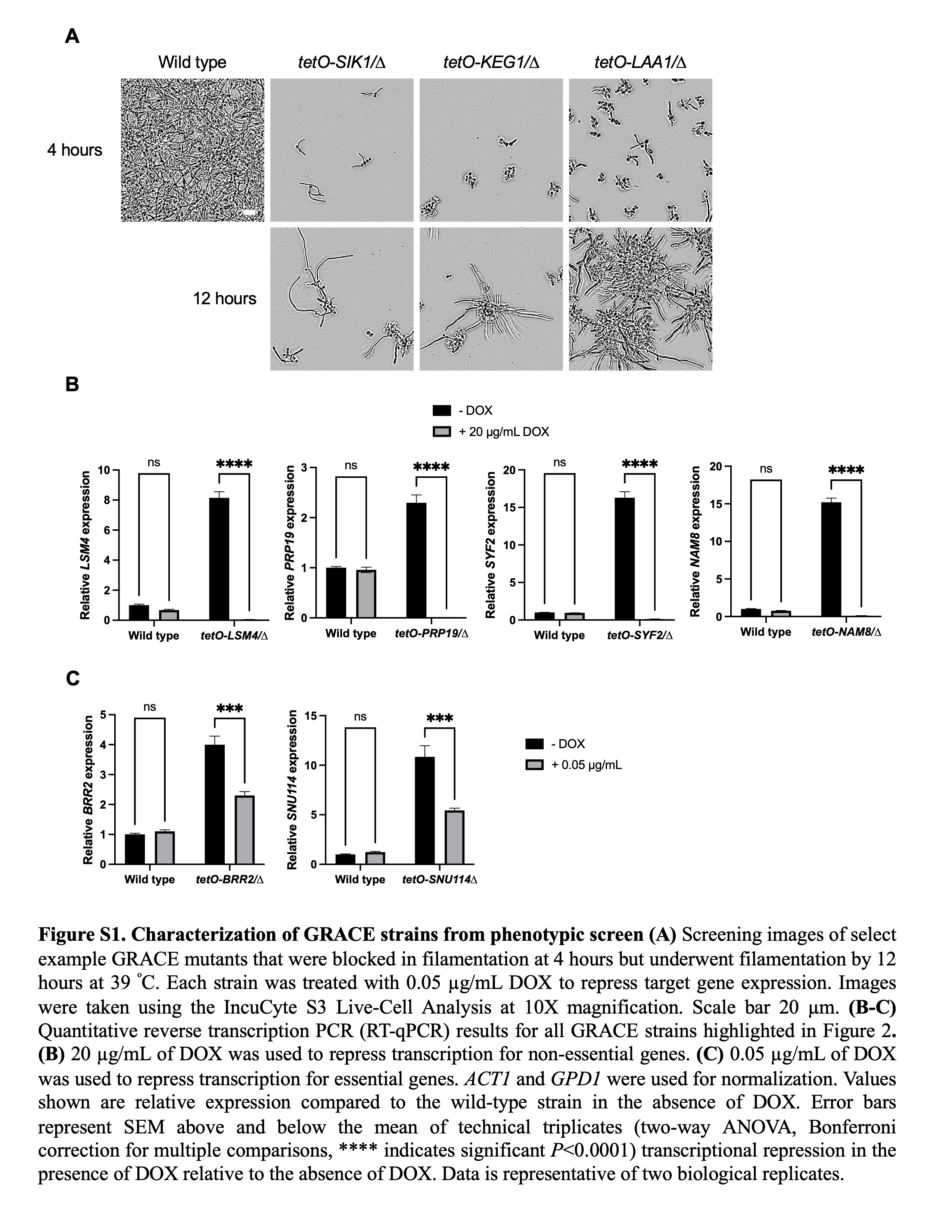

Supplement: Fig. S1 — Characterization of GRACE strains from phenotypic screen. [file mbio.01535-24-s0001.tiff]

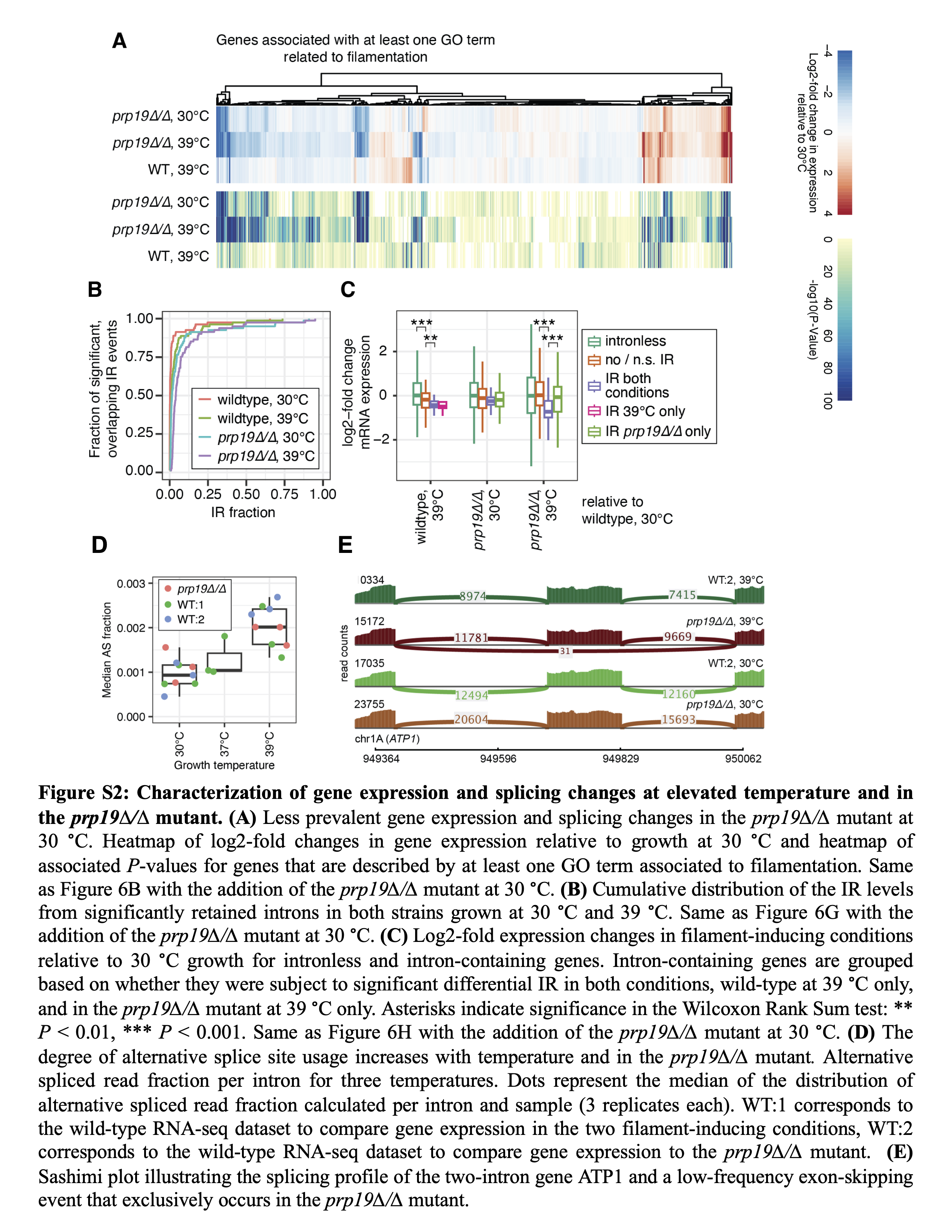

Supplement: Fig. S2 — Characterization of gene expression and splicing changes at elevated temperature and in the prp19∆/∆ mutant. [file mbio.01535-24-s0002.tiff]
