# Supplementary material for: The spliceosome impacts morphogenesis in the human fungal pathogen Candida albicans
Source: mBio. 2024 Jul 9;15(8):e01535-24. doi: 10.1128/mbio.01535-24 (PMC11323467; doi:10.1128/mbio.01535-24)
Supplement: Fig. S3 — Gene expression downstream of the TOR signaling cascade is altered in the prp19∆/∆ mutant, but Tor1 activation is not upregulated. [file mbio.01535-24-s0003.pdf]

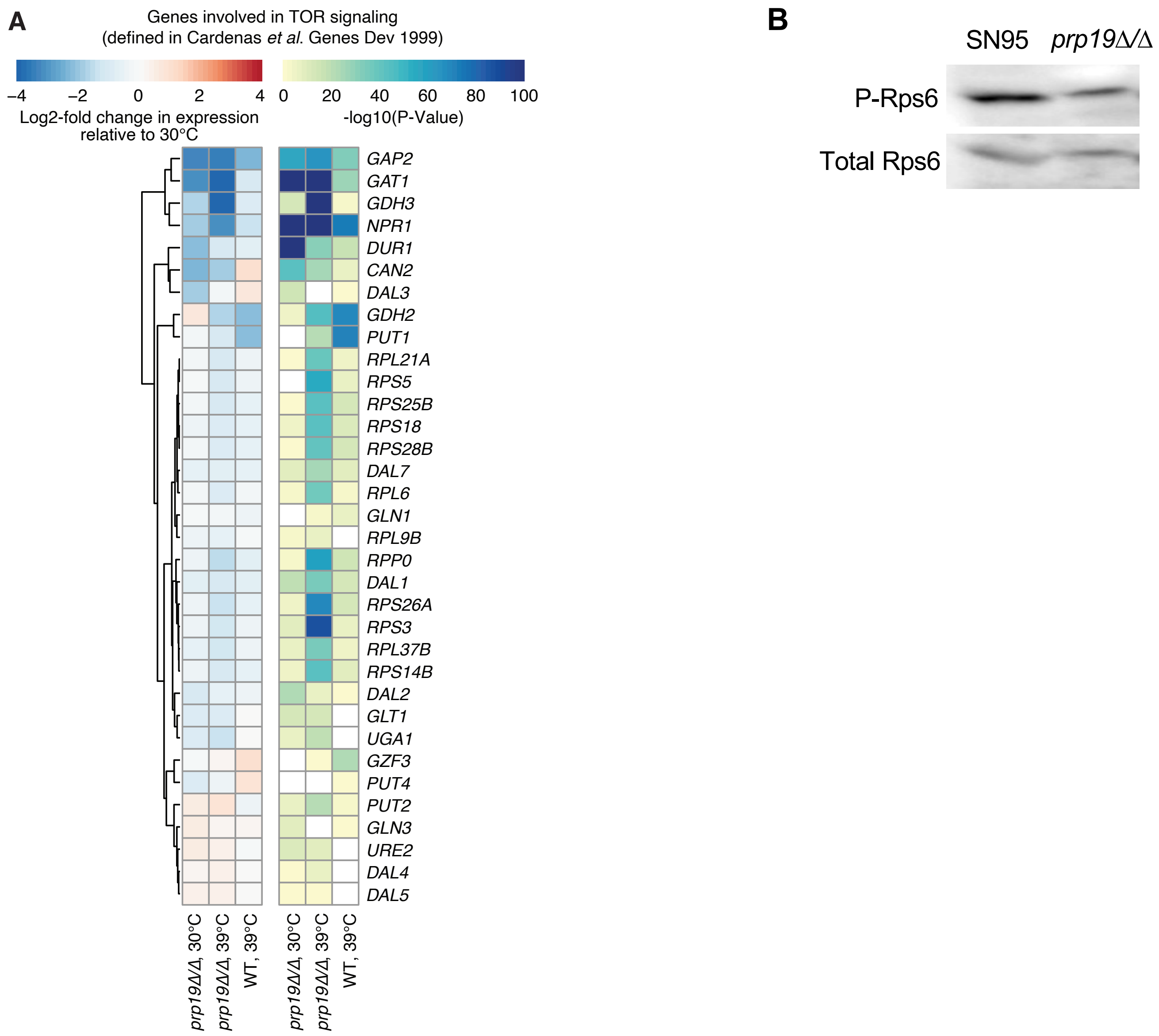

Figure S3. Gene expression downstream of the TOR signaling cascade is altered in the *prp19Δ/Δ* mutant but Tor1 activation is not upregulated. (A) Heatmap of log2-fold changes in gene expression relative to growth at 30 °C (upper panel) and heatmap of associated *P*-values for genes identified in (41). **(B)** Lysate from indicated cells grown for 1 hour at 39 °C was probed for phosphorylated (P-) Rps6 (top) and total Rps6 (bottom) as a loading control.
