# Supplementary material for: The spliceosome impacts morphogenesis in the human fungal pathogen Candida albicans
Source: mBio. 2024 Jul 9;15(8):e01535-24. doi: 10.1128/mbio.01535-24 (PMC11323467; doi:10.1128/mbio.01535-24)
Supplement: Table S6 — Strains and plasmids used in this study. [file mbio.01535-24-s0009.docx]

**Table S6. Strains and plasmids used in this study.**

| **Strain ID** | **Description** | **Genotype** | **Source** |
| --- | --- | --- | --- |
| **CaLC239** | SN95 | *arg/arg4 his1/his1 URA3/ura3::imm^434^ IRO1/iro1::imm^434^* | (Noble and Johnson, 2005) |
| **CaLC7774** | SN95 *prp19∆/prp19∆* | *arg/arg4 his1/his1 URA3/ura3::imm^434^ IRO1/iro1::imm^434^ prp19::FRT/prp19::FRT* | This study |
|  |  |  |  |
| **CaLC8643** | SN95 *ACT1(∆11-668)/ACT1(∆11-668*) | *arg4 /arg4 his1/his1 URA3/ura3::imm434 IRO1/iro1::imm434 pACT1-ACT1(∆11-668)/ pACT1-ACT1(∆11-668)* | This study |
|  |  |  |  |
| **CaLC8643** | SN95 *prp19∆/prp19∆* *ACT1*(∆11-668)/*ACT1*(∆11-668) | *arg4 /arg4 his1/his1 URA3/ura3::imm434 IRO1/iro1::imm434 prp19::FRT/prp19::FRT pACT1-ACT1(∆11-668)/ pACT1-ACT1(∆11-668)* | This study |
|  |  |  |  |
| **CaLC6106** | *C. albicans* CaSS1 (GRACE library parent) | *ura3::imm434/ura3::imm434 his3::hisG/his3::hisG leu2::tetRGAL4AD-URA/LEU2* | (Roemer *et al.*, 2003) |
| **GRACE strain, *PRP19*** | *tetO-PRP19/prp19Δ* | As CaSS1, *SAT1::tetO-PRP19/PRP1919::HIS3* | (Roemer *et al.*, 2003) |
| **GRACE strain, *SNU114*** | *tetO-SNU114/snu114Δ* | As CaSS1, *SAT1::tetO-SNU114/SNU114::HIS3* | (Roemer *et al.*, 2003) |
| **GRACE strain, *LSM4*** | *tetO-LSM4/lsm4Δ* | As CaSS1, *SAT1::tetO-LSM4/LSM4::HIS3* | This study |
| **GRACE strain, *BRR2*** | *tetO-BRR2/brr2Δ* | As CaSS1, *SAT1::tetO-BRR2/BRR2::HIS3* | (Roemer *et al.*, 2003) |
| **GRACE strain, *SYF2*** | *tetO-SYF2/syf2Δ* | As CaSS1, *SAT1::tetO-SYF2/SYF2::HIS3* | This study |
| **GRACE strain, *NAM8*** | *tetO-NAM8/nam8Δ* | As CaSS1, *SAT1::tetO-NAM8/NAM8::HIS3* | This study |
| **GRACE strain, *IST3*** | *tetO-IST3/IST3∆* | As CaSS1, *SAT1::tetO-IST3/IST3::HIS3* | This study |
| **GRACE strain, *SMX2*** | *tetO-SMX2/smx2* | As CaSS1, *SAT1::tetO-SMX2/SMX2::HIS3* | This study |
| **GRACE strain, *BUD13*** | *tetO-BUD13/bud13* | As CaSS1, *SAT1::tetO-BUD13/BUD13::HIS3* | This study |
| **GRACE strain, *MSL1*** | *tetO-MSL1/msl1∆* | As CaSS1, *SAT1::tetO-MSL1/MSL1::HIS3* | This study |
| **GRACE strain, *MUD2*** | *tetO-MUD2/mud2Δ* | As CaSS1, *SAT1::tetO-MUD2/MUD2::HIS3* | This study |
| **GRACE strain, *CUS2*** | *tetO-CUS2/cus2Δ* | As CaSS1, *SAT1::tetO-CUS2/CUS2::HIS3* | This study |
| **GRACE strain, *DRN1*** | *tetO-DRN1/drn1Δ* | As CaSS1, *SAT1::tetO-DRN1/DRN1::HIS3* | This study |
| **GRACE strain, *PRP5*** | *tetO-PRP5/prp5Δ* | As CaSS1, *SAT1::tetO-PRP5/PRP5::HIS3* | This study |
| **GRACE strain, *SLU7*** | *tetO-SLU7/slu7Δ* | As CaSS1, *SAT1::tetO-SLU7/SLU7::HIS3* | This study |
| **GRACE strain, *C5*_*04610W*** | *tetO-C5_04610W/C5_04610WΔ* | As CaSS1, *SAT1::tetO-C5_04610W/C5_04610W::HIS3* | This study |
| **GRACE strain, *LSM7*** | *tetO-LSM7/lsm7Δ* | As CaSS1, *SAT1::tetO-LSM7/LSM7::HIS3* | This study |
| **GRACE strain, *PRP8*** | *tetO-PRP8/prp8Δ* | As CaSS1, *SAT1::tetO-PRP8/PRP8::HIS3* | This study |
| **GRACE strain, *CWC21*** | *tetO-CWC21/cwc21Δ* | As CaSS1, *SAT1::tetO-CWC21/CWC21::HIS3* | This study |
| **GRACE strain, *LSM8*** | *tetO-LSM8/lsm8Δ* | As CaSS1, *SAT1::tetO-LSM8/LSM8::HIS3* | This study |
| **GRACE strain, *CWC15*** | *tetO-CWC15/cwc15Δ* | As CaSS1, *SAT1::tetO-CWC15/CWC15::HIS3* | This study |
| **GRACE strain, *LIN1*** | *tetO-LIN1/lin1Δ* | As CaSS1, *SAT1::tetO-NAM8/NAM8::HIS3* | This study |
| **GRACE strain, *HUB1*** | *tetO-HUB1/hub1Δ* | As CaSS1, *SAT1::tetO-HUB1/HUB1::HIS3* | This study |
| **GRACE strain, *SMX3*** | *tetO-SMX3/smx3Δ* | As CaSS1, *SAT1::tetO-SMX3/SMX3::HIS3* | This study |
| **GRACE strain, *DBR1*** | *tetO-DBR1/dbr1Δ* | As CaSS1, *SAT1::tetO-DBR1/DBR1::HIS3* | This study |
| **GRACE strain, *LSM6*** | *tetO-LSM6/lsm6Δ* | As CaSS1, *SAT1::tetO-LSM6/LSM6::HIS3* | This study |
| **CaLC8555** | CaLC7774 *nrg1∆/nrg1∆* | As CaLC8555 *nrg1::NAT/nrg1::NAT* | This study |
| **CaLC5368** | SN95 *nrg1∆/nrg1∆* | arg4∆/arg4∆ his1∆/his1∆ URA3/ura3::imm434 IRO1/iro1::imm434 nrg1::NAT/nrg1::NAT | (Polvi *et al.*, 2019) |
| **pLC49** | p863 (for gene disruption with *FLP-NAT*) |  | (Shen *et al.*, 2005) |
| **pLC963** | pV1393-1 (CaCas9/sgRNA entry expression vector, contains NatR gene, targeting *NEUT5L* |  | (Veri *et al.*, 2018) |
| **pLC620** | NAT-ACT1-3xFLAG, ampR (pLC49) |  | (Shapiro *et al.*, 2012) |
| **pLC763** | Ca-FLP-NAT-*tetO* |  | (Hossain *et al.*, 2020) |
| **pLC1075** | pLC963+NRG1 sgRNA |  | (Polvi *et al.*, 2019) |

**References**

Hossain, S., Veri, A.O., and Cowen, L.E. (2020) The proteasome governs fungal morphogenesis via functional connections with Hsp90 and camp-protein kinase a signaling. *mBio* **11**: e00290-20.

Noble, S.M., and Johnson, A.D. (2005) Strains and strategies for large-scale gene deletion studies of the diploid human fungal pathogen *Candida albicans*. *Eukaryot Cell* **4**: 298–309.

Polvi, E.J., Veri, A.O., Liu, Z., Hossain, S., Hyde, S., Kim, S.H., *et al.* (2019) Functional divergence of a global regulatory complex governing fungal filamentation. *PLoS Genet* **15**: e1007901.

Roemer, T., Jiang, B., Davison, J., Ketela, T., Veillette, K., Breton, A., *et al.* (2003) Large-scale essential gene identification in *Candida albicans* and applications to antifungal drug discovery. *Mol Microbiol* **50**: 167–181.

Shapiro, R.S., Zaas, A.K., Betancourt-Quiroz, M., Perfect, J.R., and Cowen, L.E. (2012) The Hsp90 co-chaperone Sgt1 governs *Candida albicans* morphogenesis and drug resistance. *PLoS One* **7**: e44734.

Shen, J., Guo, W., and Köhler, J.R. (2005) CaNAT1, a heterologous dominant selectable marker for transformation of *Candida albicans* and other pathogenic *Candida* species. *Infect Immun* **73**: 1239–1242.

Veri, A.O., Miao, Z., Shapiro, R.S., Tebbji, F., O’Meara, T.R., Kim, S.H., *et al.* (2018) Tuning Hsf1 levels drives distinct fungal morphogenetic programs with depletion impairing Hsp90 function and overexpression expanding the target space. *PLoS Genet* **14**: e1007270.
